# Determinants of protein corona adsorption and abundance revealed by interpretable machine learning across nanoparticle systems

**DOI:** 10.1038/s41598-026-55258-6

**Published:** 2026-05-30

**Authors:** Keyuan Li, Alexa Canchola, Fan Zhang, Wei-Chun Chou

**Affiliations:** 1 Environmental Toxicology Graduate Program, College of Natural & Agricultural Sciences, University of California, Riverside, CA 92521 USA; 2 Department of Environmental Sciences, College of Natural & Agricultural Sciences, University of California, Riverside, CA 92521 USA; 3 Department of Pharmaceutics, College of Pharmacy, University of Florida, Gainesville, FL 32610 USA

**Keywords:** Biotechnology, Chemistry, Materials science, Nanoscience and technology

## Abstract

Nanoparticles (NPs) hold significant potential in biotechnology, including molecular sensing, controlled release systems, and therapeutic applications. However, their behavior in biological environments remains difficult to predict because proteins rapidly absorb onto NP surfaces, forming a protein corona (PC) that reshapes their surface properties and determines their biological identity, transport, and cellular interactions. In this study, we developed large-scale deep neural network (DNN) models to predict both protein adsorption (binary classification) and relative protein abundance (regression) on NP surfaces. We utilized a well-curated and comprehensive PC dataset comprising data from 83 peer-reviewed studies, 817 NP–PC samples, and 2,497 proteins, substantially expanding the scale and diversity compared with prior studies. Then, we employed a prevalence-based filtering strategy to mitigate sparsity and batch noise and trained over 200 machine learning models across proteins. The adsorption classification models achieved high discriminative performance (AUC = 0.96), while the abundance models achieved a pooled R² of 0.67 and an average per-protein R^2^ of 0.40 on the test set. SHapley Additive exPlanations (SHAP) revealed that adsorption was predominantly governed by NP material class and surface chemistry, whereas abundance was more strongly influenced by experimental handling and kinetic parameters, particularly isolation and incubation time. Incorporation of applicability domain (AD) analysis enabled identification of reliable prediction regions, with in-AD predictions demonstrating higher confidence and reduced error for both tasks. Together, these results demonstrate that our DNN models can identify predictive drivers of PC composition and reveal feature associations consistent with patterns reported in prior mechanistic literature, offering a data-driven reference to inform nanomaterial design for biomedical and environmental applications.

**Supplementary Information:**

The online version contains supplementary material available at 10.1038/s41598-026-55258-6.

## Introduction

Nanoparticles (NPs) have emerged as a powerful platform for modern drug delivery, enabling prolonged systemic circulation, controlled and stimuli-responsive release, and tissue- or cell-specific targeting to improve therapeutic efficiency and minimize off-target effects^1^. Through rational engineering of physicochemical properties, such as size, surface charge/zeta potential, shape, core material/coating (e.g., PEGylation, ligands), and surface functionality, NPs can be tuned for multifunctionality, including optimized drug loading, pharmacokinetics, and microenvironment-selective accumulation^1–3^. However, upon exposure to biological fluids, NPs are immediately surrounded by a layer of adsorbed biomolecules, predominantly circulating proteins, forming what is known as the protein corona (PC)^4^. The PC has its own distinct physicochemical properties and biological interactions, redefines its biological identity, and governs downstream interactions such as cell targeting and uptake^5–8^, biodistribution^8,9^, immune responses^6^, drug release^9,10^, and potential off-target interactions. Thus, understanding and predicting PC composition, including protein adsorption, which reflects the thermodynamic propensity of proteins to associate with NP surfaces, and protein abundance within the corona, which additionally reflects kinetic and experimental factors influencing the measured protein profile, has become essential for the rational design of NPs in biomedical applications. Traditionally, the influence of NP properties and the PC on performance has been evaluated through trial-and-error experimentation across formulations and assay conditions, a strategy that is labor-intensive and difficult to generalize.

Mapping NP properties to PC adsorption and observed protein abundance remains fundamentally challenging because PC formation arises from a complex and nonlinear interplay among NP physicochemical attributes, experimental conditions, and intrinsic protein characteristics^11–16^. Even minor changes in incubation conditions, isolation procedures, or NP surface characteristics can markedly alter protein adsorption/ abundance profiles and downstream biological outcomes^16,17^. Such sensitivity makes traditional trial-and-error experimentation difficult to scale and limits its scalability and reproducibility. Machine learning (ML) models offer a promising alternative by capturing their nonlinear relationships and learning predictive patterns from heterogeneous datasets, and previous efforts have demonstrated feasibility for predicting PC composition^11–13,16,17^, binding affinity^14,15,18^, or cellular association^19,20^. However, many existing models rely on incomplete representations of experimental context, struggle with zero inflation (i.e., an overabundance of zero-valued protein measurements) and batch effects, and often focus on a relatively narrow subset of proteins. These challenges further complicate the joint modeling of adsorption propensity and experimentally observed abundance, which arise from related but distinct underlying processes. Moreover, few provide explicit characterization of prediction reliability through uncertainty estimation or applicability domain (AD) analysis, which constrains interpretability and practical use in decision-making^21^. These limitations highlight the need for comprehensive, context-aware, and AD-aware predictive frameworks that can scale across diverse proteins and NP systems.

To address these challenges, we developed a deep neural network (DNN) framework for large-scale prediction of both protein adsorption and relative protein abundance on the NP surface. Built upon a comprehensive PC dataset (PC-DB)^22^, our curated and comprehensive PC database, the framework integrates NPs’ physicochemical descriptors with experimental metadata to capture nonlinear interactions of PC formation. A prevalence-based filtering strategy mitigates sparsity and batch variability, enabling robust protein-specific modeling. The unified DNN architecture supports high-resolution prediction while maintaining biological interpretability. To identify predictive drivers and explore mechanism-related feature associations, we incorporated SHapley Additive Explanations (SHAP) to quantify the influence of NP features and experimental conditions on model outputs. Additionally, we implemented task-appropriate applicability domain (AD) analyses, using *k*-nearest neighbor distance for adsorption classification and leverage-based diagnostics for the abundance regression model, to characterize the reliability and confidence of predictions across feature space. Together, this study establishes a large-scale, interpretable, and AD-aware computational framework for predicting PC composition, providing quantitative insights into nano-bio interactions and supporting rational nanomaterial design.

## Results

### Overview of database

#### NP physicochemical properties and experimental conditions

**Fig. 1:**
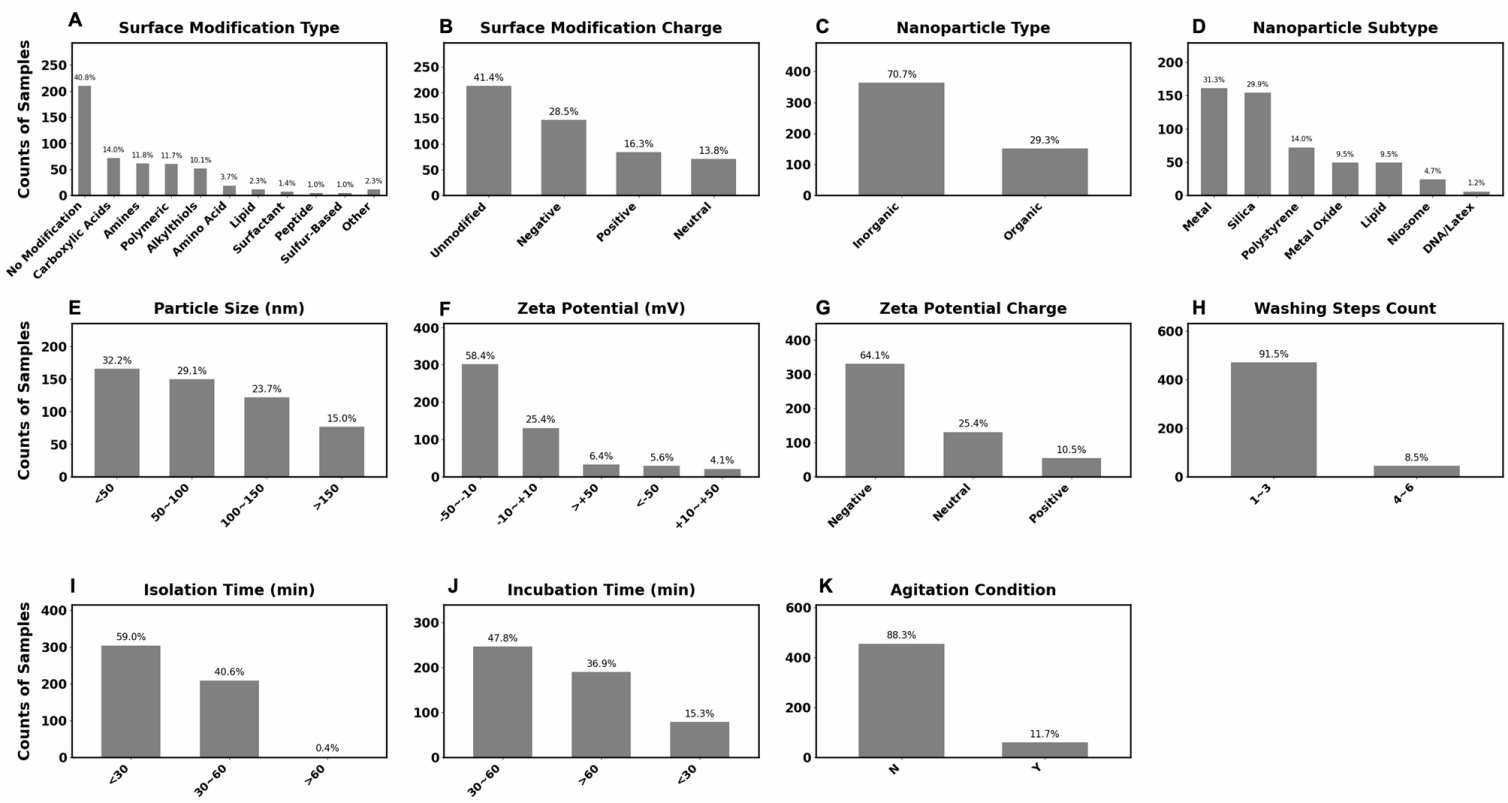
Distributions of NP properties and experimental conditions in the curated database. This figure summarizes the categorical distributions of the 11 physicochemical and experimental variables used for model development. NP physicochemical descriptors include: (**A**) Surface Modification Type, (**B**) Surface Modification Charge, (**C**) Nanoparticle Material Type, (**D**) Nanoparticle Subtype, (**E**) Particle Size (nm), (**F**) Zeta Potential (mV), and (**G**) Zeta Potential Charge. Experimental conditions are shown in panels (H–K), including (**H**) Number of Wash Steps, (**I**) Isolation Time (min), (**J**) Incubation Time (min), and (**K**) Agitation Condition. Bars represent the number of samples within each category, with percentages displayed above the bars.

The curated database used for model development contains 597 NP samples: each annotated with 11 physicochemical and experimental descriptors (Fig. 1). The workflow for protein corona (PC) database curation is illustrated in **Fig. ****S1**. These features capture the diversity of NP surface chemistry, material composition, charge characteristics, and the experimental workflows used for PC formation. Surface modification types (Fig. 1A) were dominated by unmodified NPs (40.8%), followed by carboxylic acid (14.0%), amine-based (11.8%), polymeric (11.7%), and alkylthiol (10.1%) modification. Rare modification types such as amino acid, lipid surfactant, peptide, sulfur-based and other types accounted for ≤ 5.5%. Surface modification charge (Fig. 1B) showed a similar pattern, with unmodified particles comprising 41.4%, followed by negative (28.5%), positive (16.3%), and neutral (13.8%) charge categories. At the material level (Fig. 1C), the dataset was enriched in inorganic NPs (70.7%), reflecting their long-standing use in corona research, whereas organic NPs constituted 29.3%. NP subtype distributions (Fig. 1D) showed that metal-based NPs were most prevalent (31.3%), followed by silica (29.9%), polystyrene (14.0%), metal oxide (9.5%), lipid (9.5%), niosomes (4.7%) and a combined DNA/Latex category (1.2%). Particle size categories (Fig. 1E) were primarily < 100 nm, with 32.2% of NPs < 50 nm and 29.1% between 50 and 100 nm; only 15% exceeded 150 nm. Zeta potentials (Fig. 1F) centered around moderately negative values, with 58.4% of samples between − 50 and − 10 mV, 25.4% between − 10 and + 10 mV, and progressively smaller fractions in the strongly positive or strongly negative ranges. When grouped by zeta potential charge (Fig. 1G), negative zeta potentials remained dominant (64.1%), followed by neutral (25.4%) and positive (10.5%). Experimental conditions also displayed consistent patterns across studies. Most studies used one to three wash steps (91.5%) (Fig. 1H), and Isolation procedures (Fig. 1I) were typically rapid (with 59% completed within 30 min and only 0.4% exceeding 60 min). Incubations were commonly performed for 30–60 min (47.8%) (Fig. 1J). Agitation was rarely applied (Fig. 1K), with 88.3% of samples incubated without agitation. The input features regarding NPs’ physicochemical properties and experimental conditions are listed in **Table ****S1**.

### Prevalence-based selected proteins and proteins categories

**Fig. 2:**
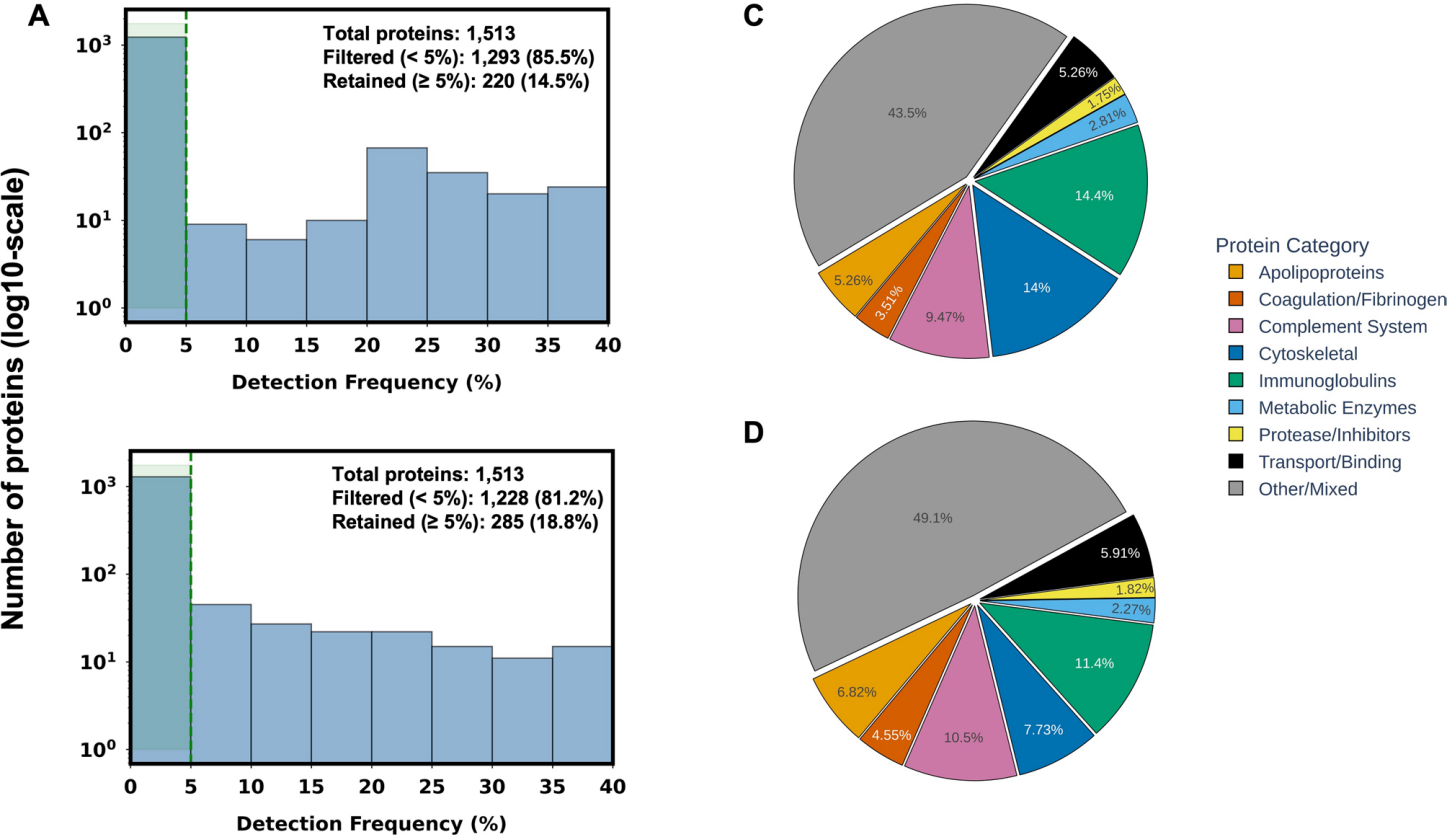
Protein selection based on detection-frequency filtering and functional category distributions of retained proteins. Detection-frequency histograms for the full protein panel used in the adsorption classification model (**A**) and abundance regression model (**B**). A 5% detection-frequency threshold (green dashed line) was applied to remove sparsely detected proteins. Functional category distributions of the retained proteins for the classification dataset (**C**) and regression dataset (**D**). Pie charts show the relative proportions of all annotated protein categories. The “Other / Mixed Function” group includes proteins that do not fall into a single dominant biological class.

We quantified detection prevalence across all 1,513 human proteins to determine which features provided sufficient coverage for reliable modeling (Fig. 2). Using a minimum detection frequency threshold of 5% non-zero values, we assessed how often each protein was detected across the samples. In the adsorption classification dataset, 81.2% of the proteins appeared in fewer than 5% of the samples, indicating a heavy-tailed distribution in which most proteins are rarely observed. After filtering, we retained 285 proteins (18.8%) for adsorption classification modeling (Fig. 2A). In the abundance regression dataset, 85.5% of proteins fell below the 5% non-zero cutoff, resulting in the retention of only 220 proteins (14.5%) (Fig. 2B). This prevalence-based filtering strategy removes extremely sparse proteins that would otherwise introduce noise and instability, while preserving a subset with sufficient empirical support to enable robust learning across samples.

To characterize the biological composition of the retained protein panels, proteins were grouped into functional categories based on curated structural and biochemical annotations (Figs. 2C-D). In both datasets, the largest fraction consisted of Other/Mixed proteins, accounting for approximately 43.5% of the adsorption dataset and 49.1% of the abundance dataset, reflecting the diverse functional roles captured in the curated protein corona datasets. Immunoglobulins constituted a substantial proportion of the adsorption dataset (14.4%) and remained prominent in the abundance panel (11.4%). Complement system proteins represented 9.47% of the adsorption dataset and 10.5% of the abundance dataset, consistent with their well-established involvement in nanoparticle recognition and immune-related interactions. Cytoskeletal proteins, including keratins, actins, and tubulins, comprised 14.0% of the adsorption dataset and 7.73% of the abundance dataset, indicating differential retention between adsorption detection and quantitative abundance modeling. Additional functional groups, including apolipoproteins (5.26% and 6.82%), transport/binding proteins (5.26% and 5.91%), coagulation/fibrinogen-related proteins (3.5% and 4.55%), metabolic enzymes (2.81% and 2.27%), and protease/inhibitor proteins (1.75% and 1.82%), each contributed smaller yet biologically meaningful fractions. Collectively, these distributions demonstrate that both protein panels encompass a broad and biologically relevant spectrum of PC components while revealing distinct compositional differences between adsorption and abundance modeling tasks. Protein identities and functional category assignments are detailed in Supplementary **Tables S2-S3**.

### Adsorption Classification Model

**Fig. 3:**
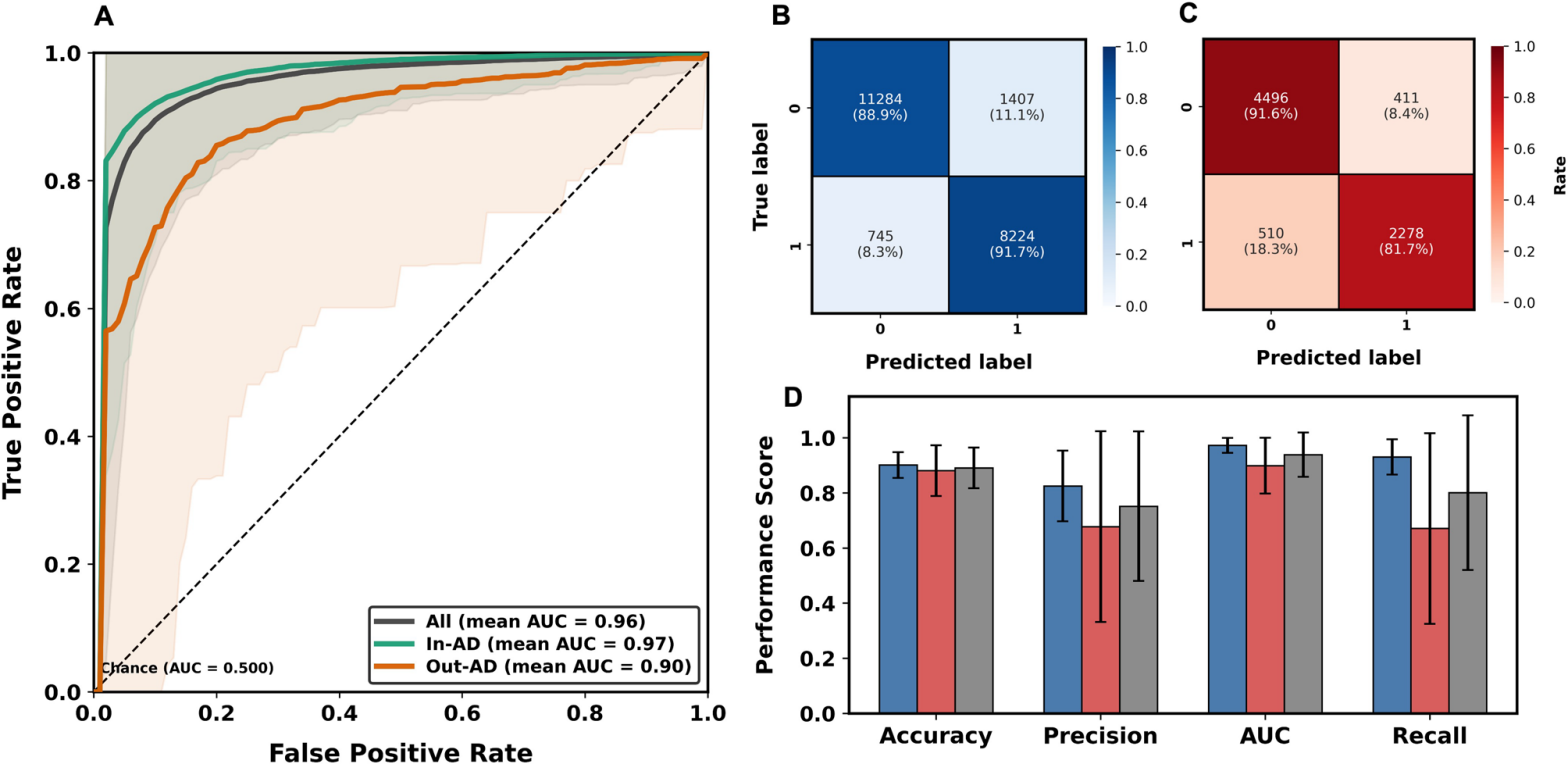
Adsorption classification model performance with AD stratification. (**A**) ROC curves for all test samples (black), in-AD samples (green), and out-of-AD samples (orange). Shaded bands around each curve represent bootstrap-derived 95% confidence intervals (gray: all; green: in-AD; orange: out-of-AD). (**B**) Confusion matrix for in-AD predictions, showing the distribution of true and false classifications. (**C**) Confusion matrix for out-AD predictions. (**D**) Summary plot of accuracy, precision, recall, and AUC across all protein classifiers, where colored bars (Blue: in-AD; Red: out-of-AD; Green: all test samples) denote the mean value and vertical error bars indicate ± 1 SD.

The adsorption classification model demonstrated strong overall discriminative performance, achieving a mean AUC of 0.96 across all proteins in the test dataset (*N* = 285 proteins). In-AD samples consistently exhibited the highest separability (average AUC = 0.97), whereas out-of-AD samples showed the expected reduction in performance (average AUC = 0.90), indicating that the prediction confidence decreased as samples deviated from the model’s applicability domain (Fig. 3A). Given the strong class imbalance in the dataset, we further evaluated model performance using precision–recall (PR) curves (**Supplementary Fig. S2**). Compared to ROC curves, PR curves can further provide an informative assessment under imbalanced conditions. The results confirm that the model maintains high precision–recall performance, while the relatively wide confidence intervals reflect variability across protein datasets, particularly for out-of-AD samples. Based on the results from confusion matrix, in-AD samples showed high predictive accuracy with 11,284 true negatives (88.9%) and 8,224 true positives (91.7%), accompanied by relatively few misclassifications (FP = 1,407, FN = 745) (Fig. 3B). In contrast, out-of-AD predictions exhibited a moderate increase in error, with 4,496 true negatives (91.6%) and 2278 true positives (81.7%), along with higher false positives (411) and false negatives (510), reflecting greater uncertainty in sparsely supported regions (Fig. 3C). Aggregate performance metrics further highlight this pattern. Across all proteins, the models achieved an overall accuracy of 0.89 ± 0.04, precision of 0.82 ± 0.10, recall of 0.88 ± 0.06, and AUC of 0.96 ± 0.02. In-AD samples consistently outperformed these averages (accuracy = 0.90, precision = 0.83, recall = 0.93, AUC = 0.97), whereas out-of-AD samples showed moderate decreases (accuracy = 0.88, precision = 0.68, recall = 0.66, AUC = 0.89) (Fig. 3D). Despite this reduction, performance remained well above random chance, indicating that the model retains meaningful predictive capability even for less familiar NPs conditions.

To complement the aggregate model evaluation, we summarized adsorption classification performance at both the overall dataset level and across functional protein categories (Table 1). The model demonstrated strong generalization, with closely aligned training and test performance across all evaluated metrics. At the overall level, training performance was high (Accuracy = 0.93 ± 0.03, F1 = 0.91 ± 0.04, Precision = 0.87 ± 0.08, Recall = 0.95 ± 0.05, AUC = 0.99 ± 0.01, MCC = 0.84 ± 0.07), with only a modest decline observed on the test set (Accuracy = 0.90 ± 0.05, F1 = 0.86 ± 0.07, Precision = 0.82 ± 0.11, Recall = 0.91 ± 0.07, AUC = 0.96 ± 0.03, MCC = 0.76 ± 0.09), indicating robust predictive stability. Category-level analysis revealed consistently strong discriminative performance across diverse functional groups, including immunoglobulins, complement system proteins, cytoskeletal proteins, and apolipoproteins, all exhibiting mean AUC values ≥ 0.94. Cytoskeletal proteins showed particularly strong performance (AUC = 0.98 ± 0.02; MCC = 0.81 ± 0.06), while immunoglobulins and complement proteins also achieved high accuracy and balanced precision-recall profiles. Although smaller categories such as protease/inhibitors and metabolic enzymes exhibited slightly higher variability, their overall performance remained comparable to larger groups.

**Table 1:** Adsorption Classification Model Performance by Overall Dataset and Protein Category.

	*N* ^b^	Acc.	F1	Precision	Recall	AUC	MCC
*Overall* ^a^							
Training	285	0.93 ± 0.03	0.91 ± 0.04	0.87 ± 0.08	0.95 ± 0.05	0.99 ± 0.01	0.84 ± 0.07
Test	285	0.90 ± 0.05	0.86 ± 0.07	0.82 ± 0.11	0.91 ± 0.07	0.96 ± 0.03	0.76 ± 0.09
*Protein Category* ^*c*^							
Apolipoproteins	15	0.89 ± 0.04	0.89 ± 0.05	0.90 ± 0.09	0.89 ± 0.04	0.94 ± 0.03	0.73 ± 0.09
Coagulation/Fibrinogen	10	0.86 ± 0.04	0.84 ± 0.07	0.81 ± 0.08	0.89 ± 0.07	0.95 ± 0.03	0.71 ± 0.09
Complement System	27	0.89 ± 0.05	0.88 ± 0.05	0.89 ± 0.08	0.89 ± 0.07	0.94 ± 0.04	0.75 ± 0.10
Cytoskeletal	40	0.93 ± 0.03	0.86 ± 0.05	0.79 ± 0.08	0.94 ± 0.08	0.98 ± 0.02	0.81 ± 0.06
Immunoglobulins	41	0.91 ± 0.04	0.88 ± 0.07	0.84 ± 0.11	0.92 ± 0.07	0.96 ± 0.03	0.79 ± 0.09
Metabolic Enzymes	8	0.87 ± 0.07	0.81 ± 0.09	0.76 ± 0.10	0.88 ± 0.10	0.95 ± 0.04	0.72 ± 0.14
Protease/Inhibitors	5	0.89 ± 0.05	0.84 ± 0.07	0.80 ± 0.12	0.90 ± 0.07	0.96 ± 0.03	0.75 ± 0.09
Transport/Binding	15	0.89 ± 0.02	0.86 ± 0.05	0.84 ± 0.10	0.88 ± 0.05	0.95 ± 0.02	0.76 ± 0.03
Other/Mixed	124	0.90 ± 0.05	0.87 ± 0.07	0.85 ± 0.12	0.90 ± 0.05	0.95 ± 0.03	0.76 ± 0.11

Note: Values represent mean ± standard deviation across proteins within each group. Performance metrics include accuracy (Acc.), F1 score (F1), precision, recall, area under the ROC curve (AUC), and Matthews correlation coefficient (MCC). Category-level metrics were calculated as the arithmetic mean ± standard deviation of the per-protein metrics within each functional category.

^a^ “Overall - Training” and “Overall – Test” summarize per-protein performance metrics computed across all proteins in the training or test sets, respectively. The calculated method was described in Supplementary section S1.2.

^b^ “N” indicates the number of proteins included in each category.

^c^ Protein category definitions are based on curated functional assignments derived from UniProt annotations and literature sources. The full list of proteins and category assignments is provided in Supplementary **Table S1**.

### Abundance regression model

A total of 220 individual abundance regression models were developed to predict the RPA on NPs surfaces, with each model targeting a distinct protein. To evaluate model performance at the protein level, we first examined the distribution of per-protein test R^2^ values (**Supplementary Fig. S3**). The results show that the majority of proteins achieved positive predictive performance, while lower overall R^2^ values were driven by a subset of proteins with limited data or higher prediction difficulty. We further performed a learning curve analysis to assess the relationship between training data size and model performance (**Supplementary Fig. S4**). The results indicate that model performance improves with increasing training data size, particularly for the test set, suggesting that predictive accuracy is partly limited by data availability.

**Fig. 4:**
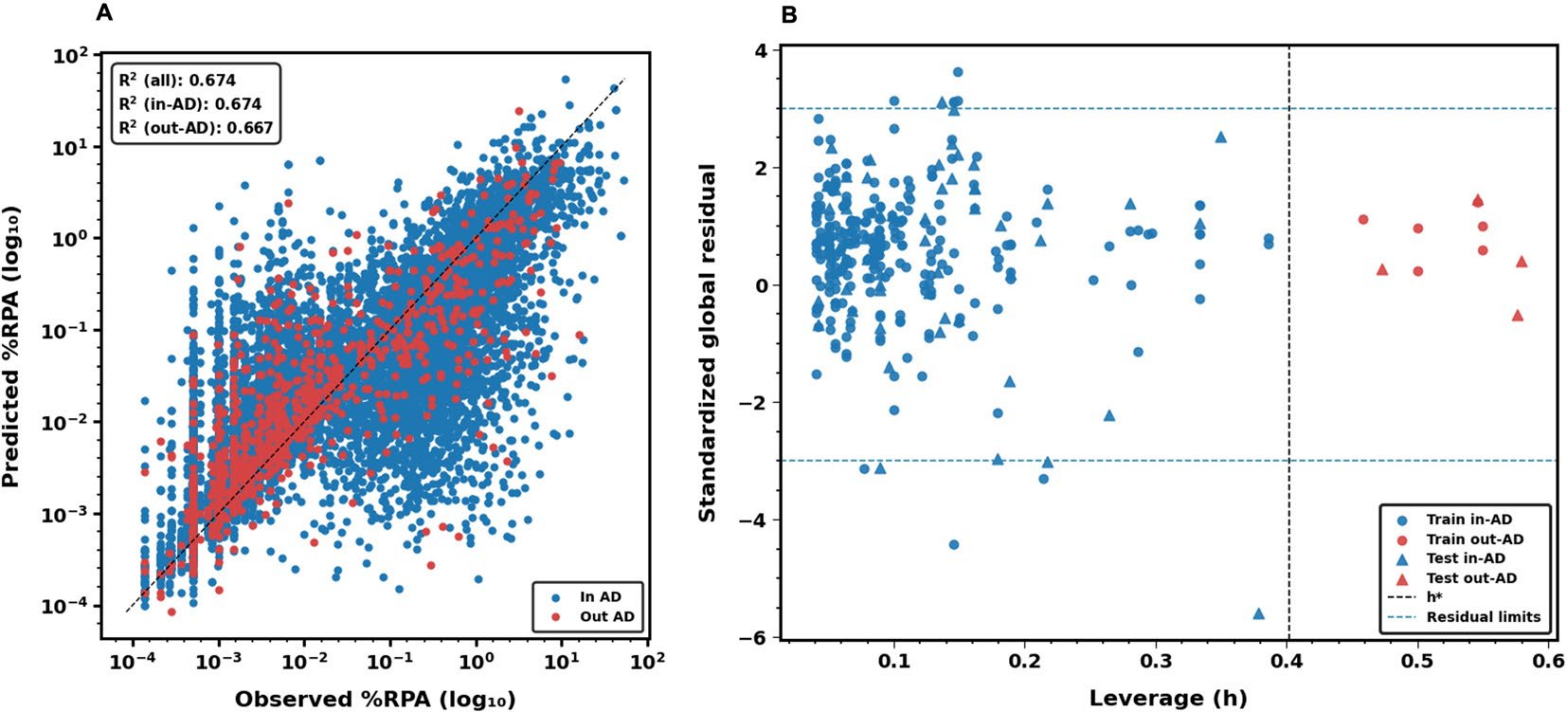
Abundance regression model performance and AD assessment. (**A**) Observed versus predicted protein abundances (log_10_ scale) across all training and test samples. Blue and red points indicate in-AD and out-of-AD predictions, respectively; dashed line represents the identity line. Inset label in the left shows the pooled coefficient of determination computed over all samples (R² all), in-AD samples only (R² in-AD), and out-of-AD samples only (R² out-AD). (**B**) William plot showing standardized global residuals versus leverage (h). Circles and triangles represent training and test samples, respectively. Horizontal dashed lines denote the ± 3σ residual threshold, and the vertical dashed line indicates the leverage cutoff h*. Points to the right of h* represent out-of-AD predictions. The circle and triangle represent the train and test samples.

When all protein-NP predictions were pooled, model outputs aligned closely with observed abundances on a log–log scale, forming a clear diagonal trend indicative of accurate magnitude estimation (Fig. 4A). AD stratification showed comparable predictive ability between in-AD and out-of-AD samples (R² = 0.67 and 0.66, respectively), and the combined dataset achieved an overall pooled R² of 0.67 (the pooled R^2^ calculation described in Supplementary Method section **S1.3**) and average per-protein R2 of 0.40. A Williams plot of standardized global residuals versus leverage further illustrated the behavior of the abundance regressors (Fig. 4B). The majority of both training and test samples fell within the accepted residual threshold (± 3σ) and below the leverage boundary h^*^. In total, 326 NP samples (94%) were classified as within the applicability domain (in-AD), while only 10 samples (2.8%) exceeded the leverage boundary and were therefore flagged as out-of-AD. Additionally, a small number of in-AD samples also fell outside the ± 3σ residual limits (9 samples, 2.6%). The overall residual distribution remained centered and symmetric, suggesting that the model’s prediction errors are unbiased across proteins of varying abundance levels.

To evaluate quantitative prediction performance, we summarized abundance regression results at both the overall dataset level and across functional protein categories (Table 2). At the overall level, where regression performance was evaluated on a per-protein basis and summarized as the mean ± standard deviation across all proteins (see Supplementary Methods Section S 1.3), the model achieved moderate explanatory power on the training set (R² = 0.63 ± 0.16), with relatively low prediction error (RMSE = 0.47 ± 0.25, MAE = 0.33 ± 0.19, MSE = 0.28 ± 0.26) on log₁₀-transformed abundance values. As expected for a heterogeneous, protein-resolved regression task, performance decreased on the test set (R² = 0.40 ± 0.33; RMSE = 0.61 ± 0.27; MAE = 0.43 ± 0.22; MSE = 0.45 ± 0.35), reflecting both reduced signal strength and increased variability across proteins while maintaining stable error magnitudes indicative of reasonable generalization. Category-level analysis revealed clear differences in quantitative predictability across protein functional classes. Cytoskeletal proteins exhibited the strongest regression performance (R² = 0.63 ± 0.17) with the lowest error metrics. Transport/binding proteins (R² = 0.49 ± 0.22) and coagulation/fibrinogen proteins (R² = 0.49 ± 0.27) also showed comparatively higher predictability, whereas complement proteins (R² = 0.32 ± 0.25), immunoglobulins (R² = 0.35 ± 0.27), and metabolic enzymes (R² = 0.18 ± 0.18) displayed lower average R² values and higher variability. Smaller categories, such as protease/inhibitors and metabolic enzymes, showed increased dispersion in performance metrics. Collectively, these results indicate that while quantitative abundance prediction is inherently more challenging than adsorption classification, the regression model captures meaningful protein-specific trends and functional-class-dependent structure in protein corona composition.

**Table 2:** Abundance Regression Model Performance by Overall Dataset and Protein Category.

	*N* ^b^	*R*²	RMSE	MAE	MSE
*Overall* ^a^					
Training	220	0.63 ± 0.16	0.47 ± 0.25	0.33 ± 0.19	0.28 ± 0.26
Test	220	0.40 ± 0.33	0.61 ± 0.27	0.43 ± 0.22	0.45 ± 0.35
*Protein Category* ^*c*^					
Apolipoproteins	15	0.42 ± 0.15	0.73 ± 0.23	0.54 ± 0.19	0.59 ± 0.38
Coagulation/Fibrinogen	11	0.49 ± 0.27	0.68 ± 0.19	0.47 ± 0.18	0.49 ± 0.26
Complement System	23	0.32 ± 0.25	0.75 ± 0.22	0.57 ± 0.23	0.61 ± 0.36
Cytoskeletal	20	0.63 ± 0.17	0.44 ± 0.17	0.27 ± 0.11	0.22 ± 0.15
Immunoglobulins	26	0.35 ± 0.27	0.58 ± 0.26	0.42 ± 0.20	0.39 ± 0.33
Metabolic Enzymes	7	0.18 ± 0.18	0.57 ± 0.21	0.39 ± 0.19	0.36 ± 0.25
Protease/Inhibitors	6	0.36 ± 0.17	0.75 ± 0.08	0.55 ± 0.15	0.57 ± 0.12
Transport/Binding	14	0.49 ± 0.22	0.68 ± 0.26	0.49 ± 0.21	0.52 ± 0.32
Other/Mixed	98	0.39 ± 0.39	0.58 ± 0.29	0.40 ± 0.23	0.43 ± 0.37

Note: Values represent mean ± standard deviation across proteins within each group. Performance metrics include the coefficient of determination (R²), root-mean-square error (RMSE), mean absolute error (MAE), and mean squared error (MSE), all computed on the log₁₀-transformed abundance scale. Category-level values were calculated as the arithmetic mean ± standard deviation of the per-protein regression metrics within each functional category.

^a^ “Overall - Training” and “Overall - Test” summarize per-protein regression performance across all proteins in the training or test datasets, respectively. The calculation procedures are described in Supplementary Methods Section **S1.3**.

^b^ “N” indicates the number of proteins included in each category.

^c^ Protein category definitions are based on curated functional annotations derived from UniProt and literature sources. The full protein list and category assignments are provided in Supplementary **Table S2**.

### SHAP-based feature importance analysis

**Fig. 5:**
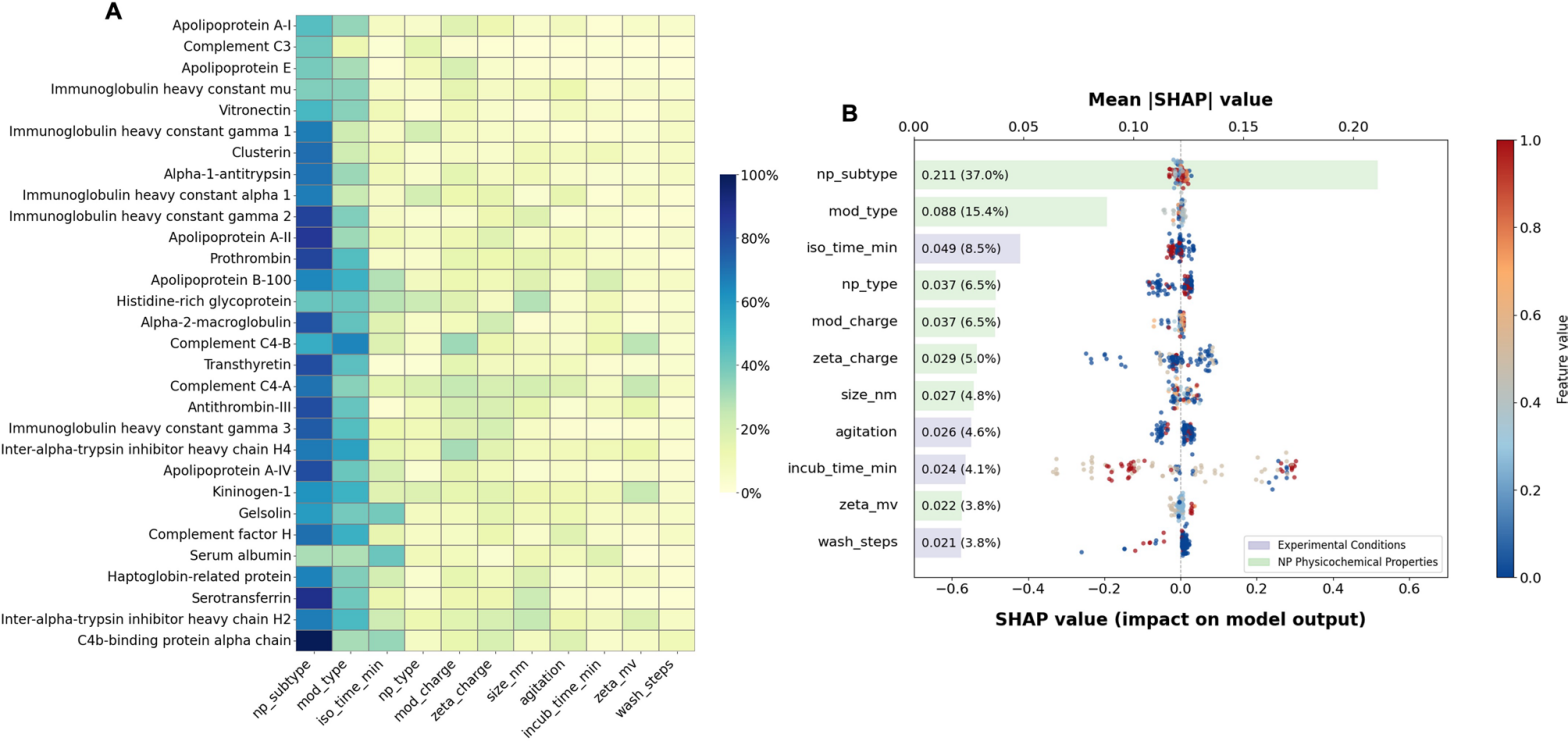
SHAP-based interpretability analysis for adsorption classification model. (**A**) SHAP heatmap showing the relative importance of 11 input features across the top 30 proteins. The x-axis represents the 11 features ranked by mean absolute SHAP values. The y-axis lists the top 30 proteins based on their expression level. The color intensity indicates the normalized average absolute SHAP value (in percentage of total), representing the contribution of a given feature to the adsorption prediction of each protein. (**B**) SHAP scatter plot for the 11 features. Bars on the left show the mean absolute |SHAP| value for each feature, with the top x-axis indicating the mean SHAP values. And the colors distinguish feature categories: NPs physicochemical properties (green) and experimental conditions (purple). Dots in the middle represent individual samples, where the bottom x-axis shows SHAP values. The color bar on the right indicates normalized feature value from low (blue) to high (red).

**Fig. 6:**
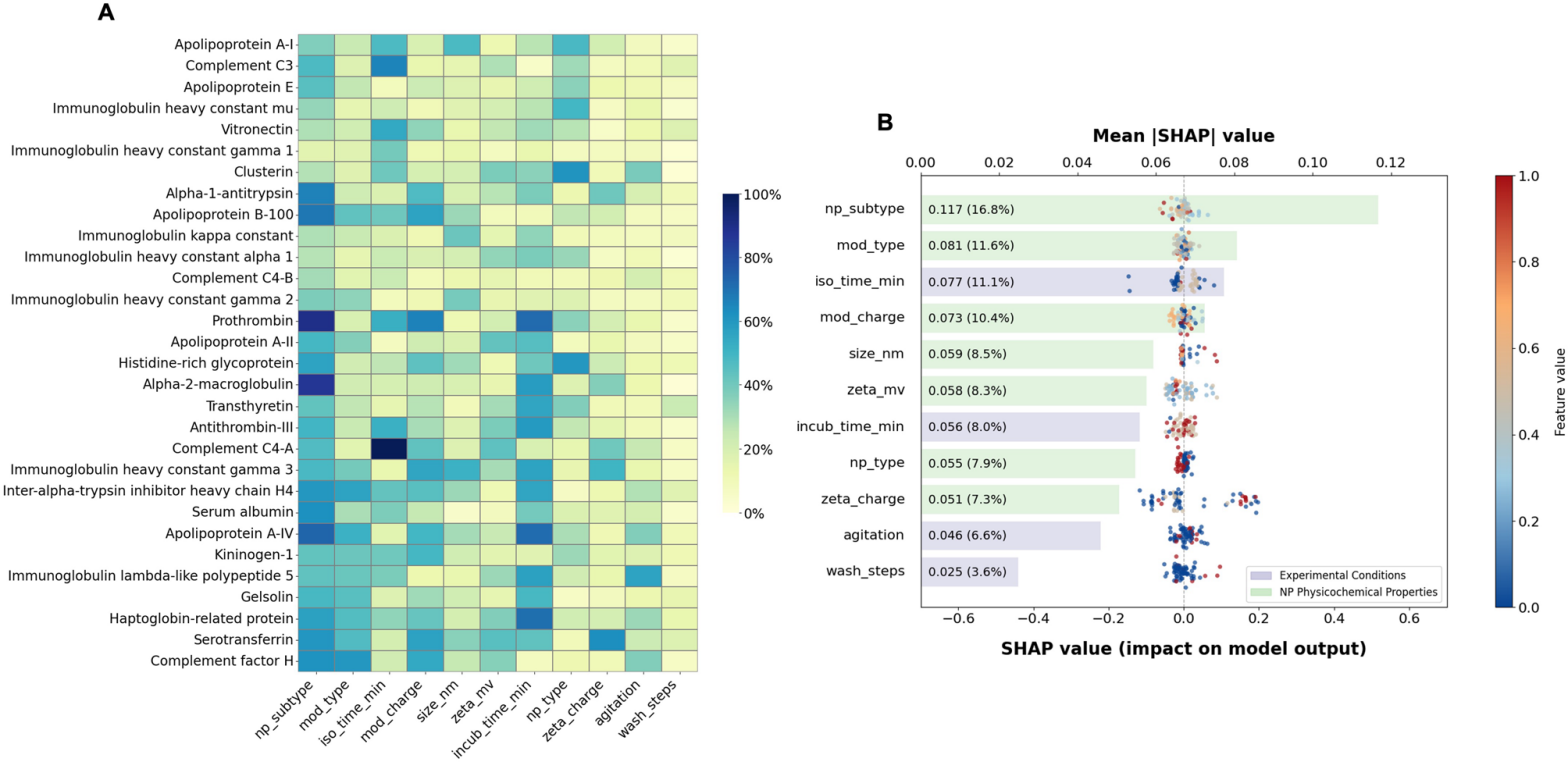
SHAP-based interpretability analysis for abundance regression model. (**A**) SHAP heatmap showing the relative importance of the 11 input features across the top 30 proteins. The x-axis represents the 11features ranked by mean SHAP values. The y-axis lists the top 30 proteins based on their expression level. The color intensity indicates the normalized average absolute SHAP value (in percentage of total), representing the contribution of a given feature to the abundance prediction of each protein. (**B**) SHAP scatter plot for the 11features. Bars on the left show the mean absolute |SHAP| value for each feature, with the top x-axis indicating the mean SHAP values. And the colors distinguish feature categories: NPs physicochemical properties (green) and experimental conditions (purple). Dots in the middle represent individual samples, where the bottom x-axis shows SHAP values. The color bar on the right indicates normalized feature value from low (blue) to high (red).

To facilitate clear visualization and interpretation, SHAP analysis was performed for all proteins and samples. SHAP was selected as it provides theoretically consistent and additive feature attributions for both global and sample-specific interpretation, enabling robust analysis of nonlinear interactions and identification of biological trends across proteins and NP conditions. Compared with local surrogate approaches such as LIME or gradient-based methods such as integrated gradients, SHAP offers greater attribution stability and direct comparability across both adsorption classification and abundance regression tasks^23^. For visualization clarity, Figs. 5 and 6 present a representative figure demonstrating the top 30 proteins and the 11 input features on adsorption classification (Fig. 5) and abundance regression (Fig. 6) models, respectively.

### Feature importance on adsorption classification model

For the adsorption classification model, SHAP values were aggregated to the parent-feature level and visualized as a heatmap (Fig. 5A). Each cell represents the mean absolute SHAP value (mean |SHAP|) of a given feature for a given protein, computed by averaging |SHAP| values across all test-set samples of that protein’s model, and subsequently normalized to the global maximum across the entire matrix and expressed as a percentage. We used mean |SHAP| rather than mean signed SHAP to avoid the ambiguity that arises when a feature has opposing directional effects across different samples, which would cause signed values to partially cancel upon averaging, understating the feature’s actual influence. Directional information is reported separately in the SHAP summary scatter plot (Fig. 5B) and in a complementary signed-SHAP heatmap (Supplementary **Fig. S5**). Figure 5A indicates that *np_subtype* showed consistently strong contributions across nearly all top 30 proteins, followed by *mod_type* and *iso_time_min*, highlight that NP core materials dominate adsorption behavior, while isolation time represents the most influential experimental condition. In particular, C4b-binding protein alpha chain, Serotransferrin, Immunoglobulin heavy constant gamma 2, Apolipoprotein A-II and Prothrombin exhibited the highest *np_subtype* contributions (> 80%), indicating that their adsorption is almost entirely governed by NP material identity. Serum albumin and Gelsolin showed relatively higher *iso_time_min* contributions compared with other proteins, suggesting that the adsorption of these two proteins is particularly sensitive to isolation time. Some proteins, including Complement C4-B, Inter-alpha-trypsin inhibitor heavy chain H4, and Histidine-rich glycoprotein, shows more distributed SHAP contributions across multiple feature categories (*np_subtype*, *mod_type*, *iso_time_min*, and several minor experimental conditions), implying that their adsorption behavior is shaped by the combined influence of NP properties and experimental conditions rather than any single dominant factor.

To further consolidate global feature importance and explore the directionality of their influence on model predictions, we generated a combined feature importance and SHAP scatter plot, as shown in Fig. 5B. Because one-hot-encoded categorical features distribute their SHAP contribution across multiple binary dummy variables, directly comparing the magnitude of individual dummy variables against continuous features can be misleading. We therefore aggregated the mean |SHAP| values of all dummy variables belonging to each parent categorical variable and report the summed contribution at the parent-feature level. Under this aggregated view, *np_subtype* emerged as the dominant predictor, accounting for 37.0% of the total SHAP magnitude, followed by *mod_type* (15.4%), *iso_time_min* (8.5%), *np_type* (6.5%), and *mod_charge* (6.5%). Together, NP physicochemical properties (*np_subtype*, *mod_type*, *np_type*, *mod_charge*, *zeta_charge*, *size_nm*, and *zeta_mv*) contributed 79% of the total SHAP magnitude, whereas experimental conditions (*iso_time_min*, *agitation*, *incub_time_min*, and *wash_steps*) contributed 21%. This support that NP physicochemical properties are the dominant determinants of whether a given protein adsorbs onto the NP surface. Beyond overall ranking, the SHAP value distributions on the right side of the plot offer insights into the direction and consistency of each feature’s effect across samples. Detailed sub-feature breakdowns for each one-hot-encoded categorical variable are provided in Supplementary **Fig. S6** and summarized below. Within *np_subtype* (**Fig. S6A**), silica NPs showed consistently high positive SHAP values, indicating that silica tends to increase the predicted probability of protein adsorption across most proteins, while metal, niosome, and metal oxide subtypes displayed predominantly negative SHAP distributions, indicating a suppressive effect on adsorption likelihood. Within *mod_type* (**Fig. S6B**), carboxylic acid and amins modifications contributed positive SHAP values, whereas lipid, sulfur-based and alkylthiol modifications shifted predictions negatively. For continuous features, *iso_time_min* exhibited a broad bidirectional SHAP distribution, consistent with its role as an important tunable experimental parameter. Other features, including *size_nm*, *zeta_mv*, *np_type*, *zeta_charge*, *agitation*, *incub_time_min*, and *wash_steps*, did not consistently increase or decrease adsorption likelihood, suggesting their influence is protein- or context-dependent rather than globally directional.

### Feature importance on abundance regression model

For the abundance regression model, SHAP values were aggregated to the parent-feature level and visualized as a heatmap (Fig. 6A), using the same calculation procedure as the classification model. A complementary signed-SHAP heatmap is provided in Supplementary **Fig. S7**. In contrast to the classification model, where *np_subtype* clearly dominated and other features showed only secondary contributions, the abundance heatmap reveals a more distributed pattern: although *np_subtype* remains as an influential feature, several other variables, including *iso_time_min*, *mod_type*, *size_nm*, *mod_charge*, and *incub_time_min*, show similar contributions across many proteins. This distribution indicates that quantitative abundance is shaped by an interplay of NP physicochemical properties and experimental conditions. Among individual proteins, Complement C4-A, Apolipoprotein A-IV, Prothrombin, and Alpha-2-macroglobulin showed particularly strong contributions from one or two features, while many other proteins exhibited more distributed SHAP patterns in multiple feature categories. A similar analysis was performed for the abundance model, and the resulting feature importance ranking with SHAP values distribution is shown in Fig. 6B. Under this aggregated view, np_subtype emerged as the top predictor, accounting for 16.8% of the total SHAP magnitude, followed by mod_type (11.6%), iso_time_min (11.1%), and mod_charge (10.4%). Together, NP physicochemical properties contributed 70.8% of the total SHAP magnitude, while experimental conditions contributed 29.3%. Compared with the classification model (78.5% vs. 21.5%), the regression model gives experimental conditions a larger predictive importance. NP properties still rank first overall, but experimental conditions catch up substantially. Isolation time alone contributes 11.1%, close to the top NP-related variables. This shift indicates that protein abundance is shaped by NP properties and experimental handling together. The SHAP value distributions on the right side of Fig. 6B further reveal directional patterns. Detailed sub-feature breakdowns for one-hot-encoded categorical variables are provided in Supplementary **Fig. S8**. For continuous features, *iso_time_min* showed predominantly positive SHAP values at lower feature values, indicating that shorter isolation times tend to increase predicted protein abundance. *size_nm* and *zeta_mv* displayed similar bidirectional patterns, with both feature values contributing both positively and negatively depending on protein context. Within *np_subtype* (**Fig. S8A**), lipid and silica NPs showed predominantly positive SHAP values, while metal oxide and polystyrene shifted predictions negatively. Within *mod_type* (**Fig. S8B**), carboxylic acid modifications contributed positive SHAP values, suggesting that this modifications tend to increase predicted abundance. Other categorical features exhibited mixed SHAP distributions with both positive and negative contributions, reflecting protein- and context-dependent effects rather than a uniform directional influence.

### Feature importance sanity check

To assess whether SHAP-derived feature importance reflects observable data patterns rather than model artifacts, we performed a stratified sanity-check analysis on representative top features (np_subtype, mod_type, and iso_time_min). Across both classification and regression tasks, the ranking of features based on raw data separation was consistent with SHAP-derived importance (Supplementary **Tables S4-S5**; see Supplementary Section S1.5 for methodological details). Specifically, np_subtype showed the largest separation in both adsorption rate and abundance, followed by mod_type and iso_time_min. These results indicate that SHAP-identified feature importance is consistent with underlying trends present in the dataset.

## Discussion

In this study, we developed and evaluated a large-scale DNN framework to predict both PC adsorption and relative abundance on NP surfaces using a curated and comprehensive PC database. Across more than 200 protein-specific models (285 adsorption classification and 220 abundance regression models), the adsorption classifiers achieved consistently high discriminative performance (AUC = 0.96 in test sets) with minimal train-test divergence and strong accuracy within the AD, indicating that the learned decision boundaries capture consistent patterns within the current dataset, with potential applicability to similar NP systems. The abundance regression model, though inherently more challenging due to continuous outcomes and greater experimental noise, still explained a substantial fraction of variance in pooled analyses, with symmetric residual distributions and Williams-plot diagnostics supporting the absence of systematic overfitting or extrapolative bias. In summary, our results integrated a curated dataset, prevalence-based protein selection, and rigorously regularized DNN architectures to enable a robust PC prediction, providing a quantitative bridge between nanomedicine design, experimental workflows, and emergent nano-bio interfaces.

### Model performance and applicability domain analysis

Both the adsorption classification and abundance regression models exhibited strong and generalizable predictive performance across a diverse panel of NP-protein interactions. For the adsorption classifier, the close alignment between training and test metrics (AUC: 0.99 ± 0.01 vs. 0.96 ± 0.03; MCC: 0.85 ± 0.07 vs. 0.79 ± 0.09; Table 1) demonstrates robust generalization and indicates that the model captures reproducible adsorption signatures rather than overfitting the training set. Category-level analysis further revealed consistently high discriminability across major functional protein classes, including immunoglobulins, complement proteins, cytoskeletal regulators, and apolipoproteins, all of which achieved mean AUC values ≥ 0.94. Cytoskeletal and immunoglobulin proteins, in particular, showed strong and balanced performance across accuracy, precision, and recall, consistent with prior studies reporting directional and material-dependent binding preferences for these protein families based on NP surface chemistry and physicochemical properties^24–26^. Importantly, these high-performance levels were accompanied by minimal train-test performance gaps and were achieved under strict controls to prevent information leakage during feature scaling, class-weighted loss function application, and hyperparameter optimization, supporting the conclusion that predictive accuracy reflects true biological signal rather than model memorization. AD analysis further corroborated classifier stability: in-AD samples consistently exhibited higher discriminability (AUC = 0.97), while out-of-AD samples showed the expected but modest reduction in performance (AUC = 0.90; Fig. 3), in agreement with established AD principles in QSAR modeling^27^. Consistent with established QSAR practice, task-specific applicability domain definitions were used to reflect differences in uncertainty structure between binary adsorption classification and continuous abundance regression, enabling prediction confidence to be evaluated in a manner appropriate to each task. Notably, even out-of-AD predictions remained well above random performance, indicating retained predictive utility for NPs with partially novel physicochemical profiles.

In contrast, quantitative abundance prediction posed a more challenging task, reflecting the continuous nature of proteomic measurements and the dynamic remodeling of the PC. At the overall level, the abundance regression models achieved moderate explanatory power on a per-protein basis (training R² = 0.63 ± 0.16; test R² = 0.40 ± 0.33), with stable error magnitudes across training and test sets (Table 2). The observed decrease in test-set R² is consistent with the inherent variability of quantitative proteomics data and differences in protein-specific binding kinetics. Category-level analysis revealed marked heterogeneity in regression performance: cytoskeletal proteins exhibited the strongest quantitative predictability (R² = 0.63 ± 0.17) with the lowest associated error metrics, while transport/binding and coagulation/fibrinogen proteins also showed relatively higher predictability. In contrast, complement proteins, immunoglobulins, and metabolic enzymes displayed lower average R² values and greater variability, consistent with their known context-dependent adsorption behavior and sensitivity to experimental conditions. AD analysis of the regression models further supported their reliability, with the majority of samples falling within the leverage threshold and predefined residual bounds, and no evidence of systematic bias across NP subtypes or experimental parameters, indicating that prediction errors primarily reflect experimental noise rather than structural model deficiencies.

Taken together, these results demonstrate that the adsorption and abundance prediction frameworks capture complementary aspects of PC formation: robust binary adsorption signatures and more nuanced, protein-specific quantitative trends. The integration of AD analysis provides an interpretable and principled means of assessing prediction confidence, highlighting the conditions under which model outputs are most reliable, which is an essential requirement for downstream applications in nanomaterial design, nano-bio interaction modeling, and risk-informed nanotechnology development.

### Model and methodological uncertainty

To evaluate the robustness of our conclusions to key methodological decisions, we examined potential sources of uncertainty associated with both model choice and protein selection strategy. These analyses were designed to determine whether the observed trends in PC adsorption and abundance are sensitive to reasonable variations in algorithmic assumptions or data filtering criteria.

### Sensitivity to model choice

Although DNNs were adopted as the primary modeling framework, we benchmarked their performance against commonly used baseline algorithms to assess whether this choice was methodologically justified (**Tables S6–S7**). For adsorption classification, the DNN achieved performance comparable to Random Forest (RF) and Logistic Regression (LR) models in terms of AUC (0.96 on the independent test set across models), while exhibiting a more balanced precision–recall profile and greater stability across proteins, as reflected by higher and more consistent MCC values. In contrast, XGBoost showed lower average performance and greater variability across all classification metrics. These results indicate that dominant adsorption signatures are relatively robust to algorithmic choice, but that the DNN provides a more consistent and scalable framework for large-scale protein-specific modeling.

More pronounced differences emerged for abundance regression. The DNN consistently outperformed all baseline regressors on the independent test set, achieving higher explanatory power (R² = 0.40 ± 0.33) and lower prediction error, whereas tree-based and linear models exhibited substantially reduced generalization and large error variance across proteins. Notably, Ridge Regression yielded negative test-set R² values (− 0.47 ± 5.99), indicating an inability to capture the nonlinear relationships governing quantitative protein accumulation. Together, these comparisons demonstrate that while binary adsorption patterns are relatively insensitive to algorithmic choice, accurate quantitative modeling of protein abundance requires flexible nonlinear architectures, supporting the selection of DNNs for abundance prediction in this work.

### Sensitivity to protein selection threshold

To assess whether the prevalence-based protein selection strategy biased model performance toward highly abundant or frequently detected proteins, we conducted a sensitivity analysis using detection-frequency thresholds of 1%, 5%, and 10% (**Tables S8–S9**). For adsorption classification, increasing the threshold from 1% to 5% substantially improved model reliability: although the 1% threshold retained more than twice as many proteins (636 vs. 285), it resulted in markedly lower F1 score (0.76 vs. 0.86) and precision (0.75 vs. 0.82), despite comparable accuracy. In contrast, classification performance at the 5% and 10% thresholds was highly consistent, with identical AUC values (0.96) and nearly identical MCC scores (~ 0.76), indicating that discriminative performance stabilized once minimal data sufficiency was achieved.

Protein prevalence exerted a stronger influence on the abundance regression model. Models trained using the 1% threshold exhibited negative test-set R² values (− 1.01 ± 7.84) with extreme variance, reflecting unstable learning driven by severe sparsity and heterogeneity in quantitative proteomic measurements. In contrast, both the 5% and 10% thresholds yielded stable and reproducible regression performance (R² ≈ 0.40–0.41) with comparable error magnitudes. Notably, increasing the threshold to 10% further reduced the number of retained proteins (173 vs. 220) without meaningful gains in predictive accuracy and with slightly increased error metrics, suggesting diminishing returns from more aggressive filtering. Collectively, these results indicate that the primary conclusions of this study are robust to reasonable variations in both model architecture and protein selection criteria and support the use of a 5% detection-frequency threshold as a principled compromise that balances protein coverage with predictive stability in our PC modeling.

### Mechanistic interpretation by SHAP analysis

The SHAP-based interpretation analysis provides interpretable feature attributions that align with mechanistic patterns reported for NP physicochemical properties and experimental conditions in PC formation. For the adsorption classification model, the strong influence of NP subtypes, particularly silica, meta, niosome, and metal oxide, is consistent with established evidence from previous studies that core material composition modulates surface energy, hydrogen-bonding capacity, and hydration shell structure, which collectively determine protein adsorption affinity^28–30^. The pronounced sensitivity of proteins such as inter-alpha-trypsin inhibitor heavy chain H1, protein AMBP, Apolipoprotein C-III, and fibrinogen beta chain to silica surfaces aligns with reports that silica promotes multilayered protein assembly through electrostatic interactions and surface silanol groups that enhance protein-surface binding^31,32^. Previous studies indicate that Fibrinogen is sensitive to silica surface and can form multilayers, while the Apolipoproteins are enriched on silica nanoparticles in a size-dependent manner^33,34^. Our multifactorial SHAP profiles further suggest that adsorption is not driven by a single physicochemical variable but instead reflects the combined influence of NP material class, physicochemical properties, and experimental conditions, consistent with literature showing that PC formation reflects multivariate and context-dependent nano-bio interactions^35,36^ and recent modeling studies^37,38^. Directionality patterns from SHAP scatter plots strengthen these mechanistic interpretations. Silica consistently increased the adsorption likelihood, whereas features such as metal, niosome, metal oxide, and sulfur-based modifications displayed predominantly negative SHAP values, suggesting inhibited binding, potentially due to tighter hydration shells, reduced conformational compatibility, or steric shielding effects introduced by surface ligands^39,40^. The mixed SHAP distributions observed for features such as size, zeta potential, and incubation conditions indicate that these variables do not exert a uniform effect on all proteins. Instead, their influence varies across protein classes, reflecting heterogeneity in protein physicochemical properties and binding preferences. This pattern is consistent with models of PC formation in which different proteins occupy distinct interaction regimes, such that a given NP feature may favor adsorption of one group of proteins while suppressing others. These divergent responses likely arise from competition, displacement dynamics, and protein-specific affinities, which together create a nonuniform and multivariate adsorption landscape.

In contrast, the abundance regression model revealed that accumulation (RPA) is modulated less by NP material type and more by experimental kinetics and physicochemical properties, consistent with dynamic PC remodeling models and the Vroman effect^41,42^. Features including isolation time, incubation time, zeta potential, particle size, and lipid-based NP subtype showed broad influence across high-abundance proteins such as apolipoproteins, complement proteins, and coagulation factors, reflecting interactions governed by exchange kinetics, competitive displacement, and retention during processing. A key directional insight from SHAP is that shorter isolation times (*iso_time_min*) consistently produced positive SHAP values, indicating higher predicted abundance. Mechanistically, shorter centrifugation/isolation steps are known to preserve loosely bound “soft corona” proteins, whereas longer or harsher isolation preferentially retains only “hard corona” proteins^26^. Similarly, lipid-based NPs displayed predominantly positive SHAP contributions, reflecting their tendency to enrich apolipoproteins and complement proteins due to hydrophobic domain exposure and membrane-mimetic surface chemistry. In contrast, features such as larger NP size and higher surface charge produced mainly negative SHAP values, suggesting decreased abundance. Larger particles exhibit lower surface-area-to-volume ratios, reducing available adsorption sites, while strongly charged surfaces may repel proteins or induce conformational rearrangements that reduce stable binding^43,44^. Mixed-direction features, including agitation and certain NP subtypes, reflect protein-specific behaviors, likely arising from variations in binding kinetics, protein structure, and competition among plasma proteins. This heterogeneity is consistent with detailed proteomic studies showing that even subtle physicochemical changes can produce divergent corona signatures across proteins^26,45^. Taken together, these results highlight a distinction in predictive drivers between adsorption and abundance predictions, consistent with mechanistic differences reported in the literature. Adsorption is driven primarily by NPs’ material identity, whereas abundance reflects the combined effects of physicochemical properties and experimental handling conditions. However, isolation time as a top predictor in the abundance model should not be interpreted simply as a biological insight. Because the curated PC database aggregates studies from different laboratories, protocol-level features such as isolation time, incubation time, and washing steps may capture not only true biological dynamics but also systematic differences in how each laboratory handles its samples^21^. As a result, their predictive contribution likely reflects a mixture of biological and protocol-driven effects. The consistency between SHAP-derived feature importance and corresponding trends observed in the raw data (Supplementary **Tables S4–S5**; see Supplementary Section S1.5) further supports that these interpretations reflect data-driven associations rather than model artifacts. While the SHAP analysis captures the combined contributions of multiple features through nonlinear model behavior, the current framework does not explicitly model interaction effects between variables (e.g., NP subtype and surface modification). As a result, potential synergistic or competitive interactions are reflected only implicitly rather than being explicitly quantified. Future work using interaction-aware modeling architectures may provide more mechanistic insight into these feature interactions.

### Limitations

While both the adsorption and abundance models showed strong predictive performance, several inherent limitations should be addressed. First, although neural networks are powerful tools for modeling nonlinear relationships, their performance is fundamentally constrained by data quality and diversity. In addition, the dataset is imbalanced toward certain nanoparticle classes (e.g., inorganic materials, metal and silica subtypes, and negatively charged particles), which may bias the learned patterns. As a result, the observed model performance may partly reflect this underlying data distribution rather than full coverage of the nanoparticle design space. In this study, many proteins appeared in only a small fraction of samples, resulting in uneven protein representation across NPs types and restricting the models’ generalization, particularly for rare or extreme protein-nanoparticle pairs. These constraints contributed to the observed performance gap between classification and regression tasks. Our classification models achieved excellent performance (AUC ~ 0.96 in test set), likely because binary adsorption patterns are relatively consistent across studies. In contrast, the regression models yielded only moderate-to-high accuracy (R² = 0.67 in pooled samples), primarily due to substantial variability in the quantification of protein abundance across different experimental platforms and protocols. The abundance values also exhibited highly skewed and long-tailed distributions, with many proteins reported at extremely low RPA levels. When the true abundance approaches zero, even small absolute deviations in prediction generate disproportionately large relative errors, resulting in these samples being poorly fitted during training and remaining inaccurate during testing. This pattern reflects both the intrinsic heterogeneity of protein adsorption and the limitations of purely data-driven models in capturing subtle differences at the low end of the abundance spectrum. Addressing these challenges through expanded and more balanced datasets, more expressive or uncertainty-aware model architectures, and integration of mechanistic or physics-informed features may substantially improve model robustness, interpretability, and applicability to complex nano-bio systems. In addition to data sparsity and imbalance, methodological heterogeneity across studies introduces further uncertainty into the regression task. Protein abundance was originally reported using a variety of quantification platforms and units (e.g., spectral counts, normalized intensity, relative ion abundance), each with its own dynamic range and sensitivity. Although these measurements were harmonized by converting all values to RPA, this normalization cannot fully compensate for intrinsic differences in detection limits, signal scaling, or quantification accuracy across methods. As a result, some variability in abundance prediction likely reflects inconsistencies in measurement rather than true biological differences. Future efforts to standardize quantification methods, adopt uniform reporting formats, or incorporate method-specific correction factors may help reduce this source of uncertainty and improve regression model reliability.

A second limitation relates to the evaluation strategy used in this study. Model performance was assessed using sample-level train-test splitting, which may overestimate generalization in multi-study datasets where samples originating from the same study share similar feature distributions. Although we explored alternative validation strategies, including study-level and grouped splitting, these approaches were not feasible due to a strong imbalance in study representation and limited sample diversity within individual studies, which would result in unstable model training and evaluation. In addition, the protein-specific modeling framework requires training independent models for approximately 200 proteins; implementing a leave-one-study-out validation across 52 studies would require ~ 10,400 models, and when combined with cross-validation and Bayesian hyperparameter optimization, this would scale to millions of training runs, making such analyses computationally prohibitive. Therefore, the reported performance should be interpreted as within-dataset evaluation rather than strict out-of-distribution generalization. Future work with larger, more balanced datasets and alternative modeling frameworks may enable more rigorous evaluation of generalizability across studies and NP systems.

A third limitation relates to dose-related features. Although the NP dose is biologically relevant, it was excluded from the final models due to its minimal contribution to predictive accuracy and substantial inconsistency across studies. To evaluate whether NP dose meaningfully influenced model outcomes, we performed SHAP-based feature importance analysis using a dataset that retained the dose variable (**Figs. S9-S10**). Across both adsorption and abundance models, NP subtype, size, and key experimental parameters (e.g., incubation time, agitation) consistently emerged as the dominant predictors, whereas the Final NP Mass (mg) feature contributed minimally. For nearly all proteins, the dose feature ranked at the bottom of importance: in classification models, it accounted for less than 2% of total contribution, and in regression models, its SHAP value was effectively zero, indicating no measurable influence on predictions. This weak contribution likely stems from several data-level issues. Incorporating the dose column substantially reduced usable sample size (from 515 to 252 in classification and from 346 to only 53 in regression), limiting the model’s ability to learn meaningful dose–response patterns. Additionally, most reported doses fell within a narrow range (0–5 mg), restricting the dynamic variation needed for quantitative modeling. Reporting inconsistencies further compounded these challenges, as dose was expressed in disparate units (e.g., mg, µg, mg/mL, molar concentration, particle count, or surface-area–based units), and essential parameters for unit conversion, such as solution volume, incubation volume, or NP density, were often missing. Although we attempted to harmonize these values to a single metric (total NP mass incubated), many entries had to be coded as “Not Reported”, preventing reliable standardization. Together, these factors explain the negligible dose contribution in the SHAP results and highlight the need for more comprehensive and standardized dose reporting to elucidate true dose-dependent effects on protein corona formation.

A fourth limitation is the use of a unified feature representation for both inorganic and organic NPs. Although these two NP classes possess fundamentally different intrinsic material properties, the relatively small number of organic NP samples in the curated dataset limited our ability to construct separate, material-specific models or to introduce chemistry-tailored descriptors for organic systems. To ensure model stability and maximize data utilization, both inorganic and organic NPs were therefore described using a common set of physicochemical descriptors and experimental condition features. Importantly, this unified representation did not appear to adversely affect model performance: both adsorption classification and abundance regression models exhibited stable predictive accuracy and minimal train-test divergence, and material subtype features (*np_subtype_Metal* and *np_subtype_Lipid* in Figs. 5 and 6) consistently ranked among the most influential predictors in SHAP analyses, indicating that material-dependent effects were learned implicitly by the models. Nevertheless, this approach may underrepresent subtle organic NP-specific interactions, such as those arising from polymer composition, lipid packing, or degradable surface chemistries, which are not fully captured by coarse physicochemical descriptors^21,46,47^. As larger and more diverse datasets for organic nanoparticles become available, future extensions of this framework could incorporate material-specific feature engineering, hierarchical or mixture-of-experts architectures, or transfer learning strategies to disentangle better inorganic- and organic-specific mechanisms of protein corona formation.

A further consequential limitation is the absence of biological fluid composition descriptors, including serum vs. plasma, and total protein concentration. PC composition is sensitive to these factors, since differences in NP incubation source (serum vs. plasma) will change the PC composition. These descriptors could not be incorporated in the present framework because the underlying PC literatures does not consistently report this information, which also identified as a key barrier to robust PC ML model development. Including biological fluid descriptors therefore represents a major direction for future PC modeling and reporting efforts.

An additional limitation is that protein intrinsic physicochemical properties (e.g., isoelectric point, hydrophobicity, molecular weight, and structural features) were not included as input features. While these descriptors are mechanistically important for protein corona formation, they are not informative within the current modeling framework because each model is trained independently for a single protein, making protein-level features constant across all samples within a given model. As a result, such descriptors do not provide learnable variation. Incorporating protein physicochemical features would require a different modeling strategy, such as a shared or dual-encoder architecture that jointly represents nanoparticle properties, experimental conditions, and protein characteristics across proteins. Future work adopting such architectures may improve both mechanistic interpretability and predictive performance. Additionally, each protein was modeled independently. In reality, PC formation is a collective and competitive process in which proteins interact with one another, compete for binding sites, displace previously adsorbed proteins, and form multilayer structures over time. By not incorporating protein-protein or cooperative adsorption dynamics, the current framework cannot fully capture the complex interplay governing corona composition. Future work that integrates joint modeling, co-adsorption patterns, network-based protein relationships, or mechanistic competitive binding models (e.g., Vroman-type dynamics) may provide a more complete representation of protein corona assembly.

Finally, our models were developed entirely using in vitro datasets, which do not fully represent the complexity of in vivo environments. Physiological factors such as dynamic blood flow, organ-level transport, diverse biomolecular compositions, and continuous protein exchange may lead to PC profiles that differ substantially from those observed in static in vitro systems. Nevertheless, the present framework can serve as a foundational model. With the availability of future in vivo datasets, transfer learning or fine-tuning approaches could be applied to adapt the model to physiological conditions and enhance its translational relevance.

## Conclusion

This work establishes a large-scale, interpretable, and AD-aware DNN for predicting PC composition that integrates curated experimental data with mechanistically meaningful NPs and assay descriptors. By resolving PC adsorption and abundance patterns across hundreds of proteins, the models uncover distinct physicochemical and experimental determinants of PC formation and offer a transparent basis for understanding nano-bio interactions. The incorporation of SHAP-based interpretation and AD analysis provides a principled means to assess prediction reliability and extract mechanistic insight, enabling both hypothesis generation and practical decision support. Beyond improving reproducibility in PC characterization, this framework lays the foundation for scalable, data-driven approaches that can guide rational NPs design, support harmonization of experimental workflows, and be extended to in vivo systems or multi-modal modeling in future studies.

### Methods

#### Data collection and curation

All data used in this study were derived from the curated Protein Corona Database (PC-DB), described in detail in our previous publication^22^. This dataset was constructed through a systematic review of 83 peer-reviewed experimental studies and combined NPs formulations, experimental conditions, and associated PC profiles. Only UniProt-reviewed proteins with valid quantification under defined incubation and centrifugation conditions were retained for modeling. The full PC-DB consists of 817 NP-PC samples, and 2,497 proteins identified across 4 different species (human, mouse, rat, and bovine). To ensure biological consistency, this study focused on human-derived PC profiles. Additionally, we restricted input variables to 11 features based on their literature-reported influence on PC composition and their broad availability across entries. These filters reduced the available dataset to 597 samples and 1,513 human proteins.

For the adsorption classification model, all available samples with binary adsorption labels were retained after removing missing values (“N/A” and “Not Reported”), resulting in 515 available samples. The abundance regression model requires consistent quantitative measurements, yet the literature reports a mix of mass-based (e.g., spectral counts) and molar-based (e.g., LFQ, iBAQ) measures that are not directly comparable. We therefore converted available measurements (mass-based unit) to Relative Protein Abundance (RPA) using a standardized procedure (Details in Supplementary, **Section S1.1**) and retained entries originally reported in RPA when conversion was not feasible. This harmonization yielded a regression dataset of 346 samples. The workflow used to filter the classification and regression dataset based on these criteria is illustrated in Supplementary **Fig. S1**.

### Protein selection and functional categorization

To ensure adequate signal robustness and reduce sparsity in model training, we applied a detection-frequency filter to all protein abundance features in both the adsorption classification and abundance regression datasets. For each protein, we computed the proportion of samples in which its measured abundance was non-zero (i.e., detection frequency). Proteins were retained for modeling only if they were detected in at least 5% of samples (i.e., non-zero abundance values were observed in ≥ 5% of all samples). This threshold was chosen to exclude proteins with highly sporadic or near-absent measurements, which are unlikely to provide a reliable predictive signal for downstream modeling.

After prevalence-based filtering, all retained proteins were assigned to functional categories to support biological interpretation. Protein categorization was performed using a rule-based mapping informed by standardized protein annotations available in UniProtKB and associated Gene Ontology (GO) biological-function terms^48^. Proteins were grouped into major functional classes, including immunoglobulins, complement system components, apolipoproteins, cytoskeletal proteins (e.g., keratins, actins, tubulins), metabolic enzymes, coagulation-related proteins, and membrane-binding or transport proteins, based on canonical names and UniProt functional descriptors. Proteins without a dominant or well-defined functional role, or those spanning multiple functional categories, were placed into an “Other / Mixed Function” category. A complete list of all retained proteins, organized by functional categories with corresponding protein names and detection counts, is provided in Supplementary **Tables S2-S3**.

#### Data preprocessing

In this study, we utilized 11 features encompassing both NPs physicochemical properties and experimental conditions, including Modification type, Modification charge, Type, Subtype, Size (nm), Zeta Potential (mV), Zeta Potential charge, Incubation time (min), Agitation During Incubation, Centrifugation time (min), and Washing steps counts (All features’ names and their abbreviations were listed in **Table ****S1**). These 11 features were selected based on their well-documented relevance to PC composition across nanomedicine studies and their consistent presence within our dataset, ensuring both biological significance and broad sample coverage for modeling.

Before model fitting, categorical features without ordinal relationships (e.g., Type, Modification charge) were transformed using one-hot encoding, while ordinal variables (e.g., Zeta Potential, Incubation Time) were first transferred to numerical groups and then encoded using label encoding. Entries containing missing values (“N/A” or “Not Reported”) were excluded to ensure dataset integrity. For the adsorption model (classification tasks), binary adsorption labels were assigned as 1 for the presence and 0 for the absence of the protein. To address class imbalance within the training data, we use a class-weighted loss function^49^ rather than data-level oversampling approaches like Synthetic Minority Over-sampling Technique (SMOTE)^50^. This choice preserves the physical and experimental integrity of the feature space, as interpolation-based oversampling can generate unrealistic representations when categorical descriptors are encoded as one-hot vectors^51^. For regression tasks, the dataset was randomly split into 80% for training and 20% for testing. Subsequently, target variables were log-transformed to stabilize variance and reduce the influence of extreme values, and then scaled to the [0–1] range using min-max normalization. These preprocessing steps improved numerical stability and facilitated faster, more consistent convergence during model training.

### Model development and evaluation

#### Neural network architecture and training strategy

A fully connected DNN architecture was applied for both adsorption classification and abundance regression tasks. Each model consisted of one input layer, two hidden layers, and one output layer, with rectified linear unit (ReLU) activations applied after each hidden layer. To improve training stability and reduce overfitting, batch normalization and dropout regularization were incorporated between layers. All models were implemented in PyTorch and trained using a unified set of NP physicochemical descriptors and experimental conditions (**Table ****S1**). Importantly, identical network architecture and training strategy were applied across all protein-specific models, enabling scalable, large-scale PC prediction while ensuring that observed performance differences reflect protein-dependent adsorption and abundance patterns rather than architectural variation. For adsorption classification, models were trained to output class logits, whereas abundance regression models produced continuous abundance predictions.

In order to improve model performance and robustness, we implemented a combined strategy of 3-fold cross-validation with early stopping and Bayesian optimization. In 3-fold cross-validation, the dataset was split into three subsets: two subsets were used for training and one for validation in each iteration, cycling through all combinations. This approach helps evaluate model generalizability across different data partitions. Early stopping was integrated during training to monitor validation performance. When no improvement was observed for a defined number of consecutive epochs (patience threshold), training was automatically terminated to prevent overfitting and unnecessary computation. To optimize the model hyperparameters, we utilized Bayesian optimization via Optuna^52^, which allows efficient exploration of the hyperparameter space. Parameters such as the number of hidden units, learning rate, dropout rate, and batch size were tuned using this approach. The optimal hyperparameter combinations identified through cross-validation were then used to retrain the final models on the entire training dataset.

#### Adsorption classification model evaluation

Each protein was modeled independently, resulting in a distinct model per protein for both the classification and regression tasks. This individualized modeling approach, following the strategy described by Fu et al. ^38^, prevents data leakage associated with duplicated nanoparticle features and enables each model to learn protein-specific adsorption patterns. Classification performance was summarized as the mean across all protein-specific classifiers, using accuracy, precision, recall, F1 score, area under the ROC curve (AUC), and Matthews correlation coefficient (MCC) as evaluation metrics. To summarize model performance at the overall level, per-protein evaluation metrics were aggregated and reported as the arithmetic mean ± standard deviation across all proteins. Metric definitions are provided in Section S1.2 of the Supplementary Methods. In addition, mean ROC curves with 95% confidence intervals were generated by averaging true positive rates across protein-specific classifiers at fixed false positive rates, and aggregated confusion matrices were constructed by concatenating prediction outcomes across all proteins to provide a global overview of classification behavior.

#### Abundance regression model evaluation

Regression model performance was assessed using the coefficient of determination (R²) and mean squared error (MSE), which measure the proportion of explained variance and the average squared deviation between predicted and observed values. All abundance values were evaluated on a log-transformed scale to ensure stable comparisons across several orders of magnitude. For each protein-specific regression model, predictions were generated independently for the training and test sets, and performance metrics were computed on a per-protein basis. Overall regression performance at the protein level was summarized as the arithmetic mean ± standard deviation of per-protein metrics across all 220 models. In addition to per-protein evaluation, pooled (global) regression performance was assessed by concatenating predicted and observed abundance values across all protein-NP pairs and computing overall R², RMSE, MAE, and MSE. This pooled analysis provides a complementary, pair-level assessment of how well the modeling framework captures global abundance magnitude across the full PC-NP dataset. Prediction errors were further expressed as standardized global residuals, calculated by dividing each raw residual by the standard deviation of all residuals across the pooled dataset. This variance-normalized representation enables scale-independent comparison of errors and supports AD analysis by identifying observations exceeding ± 3 standardized units. Leverage values were computed for each sample, and Williams plots were used to jointly evaluate leverage and standardized residuals, enabling identification of extrapolative predictions and extreme-error cases. Definitions of all regression performance metrics and AD thresholds are provided in Section S1.3 of the Supplementary Methods.

### Applicability domain analysis

Because the abundance regression and adsorption classification models differ in outcome structure and error behavior, task-specific AD definitions were applied, using distance-based criteria for classification and leverage- and residual-based criteria for regression. All AD assessments were performed using the same standardized NP feature matrix to ensure comparability across models.

### AD for the adsorption classification model

For the binary protein-adsorption classification task, we applied a feature-space distance-based AD using a k-nearest neighbors (k-NN) approach^53^. For each training sample $\:{x}_{i}$, we computed Euclidean distance to the *k* nearest neighbors (excluding self-distance) and defined the mean k-NN distance as:

$${\overline{d}}_{k}\left({x}_{i}\right)=\frac{1}{k}\sum_{{x}_{j}\in\:{N}_{k}\left(i\right)}||{{x}_{i}-{x}_{j}||}_{2}$$

With k = 5. The distribution of $\:{\overline{d}}_{k}\left({x}_{i}\right)$ across all training samples was used to determine AD threshold. A sample was considered outside the AD (i.e., out-of-AD) if:

$$\:{\overline{d}}_{k}\left({x}_{i}\right)>\tau\:,\:\:\:\:\:\:\:\:\tau\:=\:{\mathrm{Q}\mathrm{u}\mathrm{a}\mathrm{n}\mathrm{t}\mathrm{i}\mathrm{l}\mathrm{e}}_{0.95}\left({\overline{d}}_{k}\left({x}_{train}\right)\right).$$

For each test sample $\:{x}_{t}$, the same average k-NN distance $\:{\overline{d}}_{k}\left({x}_{t}\right)$ was computed, and samples satisfying $\:{\overline{d}}_{k}\left({x}_{t}\right)>\tau\:$ were classified as within the AD. This k-NN AD provides a geometric criterion that identifies test NPs whose physicochemical feature profiles lie in sparsely populated regions of the training feature space. The AD mask was applied to generate in-AD vs. out-of-AD ROC curves and AD-filtered probability outputs.

### AD for the abundance regression model

For the continuous PC-abundance prediction model, we quantified prediction reliability using the leverage (hat-matrix) method, which is widely used in Quantitative Structure-Activity Relationship regression^54^ to assess whether a test point is extrapolative relative to the training domain. For the standardized feature matrix *X*, the hat matrix *H* was computed as:

$$\:H=X{\left({X}^{\mathrm{T}}X\right)}^{-1}{X}^{\mathrm{T}},$$

The leverage values for each training sample $\:{x}_{i}$ is: $\:{h}_{i}\:=\:{H}_{ii}$. The threshold for excessive leverage was defined as h^*^ = 3(*p* + 1)/*n*, where *p* is the number of features and *n* is the number of training samples. Standardized global residuals were obtained by aggregating model residuals across all proteins and normalizing them by training residual variance. Test samples were considered outside the AD if they exceeded either the leverage threshold ($\:{h}_{t}>{h}^{\mathrm{*}}$) or a standardized residual magnitude of three ($\:\mid\:{r}_{t}\mid\:>3$).

### Interpretability and feature importance analysis

To interpret the trained models and quantify the influence of each input variable, we applied SHAP (SHapley Additive exPlanations) analysis^23^. SHAP is a game-theoretic framework that attributes a contribution value to each feature, representing its effect on the model output relative to the average prediction. This approach enables both local interpretability (per sample) and global interpretability (across all samples), making it suitable for complex biological datasets with interdependent variables. For each protein-specific model, SHAP values were computed to estimate the impact of NPs physicochemical properties and experimental features on the predicted outcomes. The results were summarized in two complementary ways. First, a heatmap of mean absolute SHAP values was generated to compare feature importance across all proteins. Second, global SHAP summary plots were produced to visualize both the magnitude and direction of each feature’s contribution. Features were ranked according to their mean |SHAP| values, highlighting dominant influenced NPs physicochemical properties (e.g., surface modification, material subtype, zeta potential) and experimental factors (e.g., incubation and isolation time).

## Supplementary Information

Below is the link to the electronic supplementary material.

Supplementary Material 1

## Data Availability

All data used in this study were derived from a curated PC-DB compiled from published experimental studies. The processed datasets used for model development include 515 NP-PC samples for the adsorption classification model and 346 samples for the abundance regression model, which are provided in the **Supplementary Excel files** . All model code, including the code scripts for developing the DNN model and for the model application, as well as data files, can also be accessed in the GitHub repository: [https://github.com/choulab210](https:/github.com/choulab210) .
