# Supplementary material for: Determinants of protein corona adsorption and abundance revealed by interpretable machine learning across nanoparticle systems
Source: Sci Rep. 2026 May 30;16:24775. doi: 10.1038/s41598-026-55258-6 (PMC13458488; doi:10.1038/s41598-026-55258-6)
Supplement: Supplementary file 1 [file 41598_2026_55258_MOESM1_ESM.docx]

**SUPPLEMENTARY INFORMATION**

**Determinants of Protein Corona Adsorption and Abundance Revealed by Interpretable Machine Learning Across Nanoparticle Systems**

Keyuan Li ^1±^, Alexa Canchola ^2±^, Fan Zhang^3^, Wei-Chun Chou^1,2,*^

^1^ Environmental Toxicology Graduate Program, College of Natural & Agricultural Sciences, University of California, Riverside, California 92521, United States

^2^ Department of Environmental Sciences, College of Natural & Agricultural Sciences, University of California, Riverside, California 92521, United States

^3^ Department of Pharmaceutics, College of Pharmacy, University of Florida, Gainesville, Florida 32610, United States

^±^ These authors contributed equally to the work

* To whom correspondence should be addressed

Phone: +1-951-827-9323, Email: [weichun.chou@ucr.edu](mailto:weichun.chou@ucr.edu) (W.-C. C.)

**Table of Contents**

[**Section S1: Methods**	3](#_Toc220160246)

[**S1.1 Unit normalization**	3](#_Toc220160247)

[**S1.2 Performance metrics for adsorption classification model**	3](#_Toc220160248)

[**S1.3 Performance metrics for abundance regression Model**	4](#_Toc220160249)

[**S1.3.1 Per-protein regression metrics**	5](#_Toc220160250)

[**S1.3.2 Pooled regression performance**	5](#_Toc220160251)

[**S1.4 Standardized Global Residuals for Error Analysis**	6](#_Toc220160252)

[**Section S2: Supplementary Tables**	7](#_Toc220160253)

[**Table S1**.	7](#_Toc220160254)

[**Table S2**.	10](#_Toc220160255)

[**Table S3**.	13](#_Toc220160256)

[**Table S4**.	14](#_Toc220160257)

[**Table S5**.	15](#_Toc220160258)

[**Table S6**.	16](#_Toc220160259)

[**Table S7**.	17](#_Toc220160260)

[**Section S3: Supplementary Figures**	18](#_Toc220160261)

[**Fig. S1.**	18](#_Toc220160262)

[**Fig. S2.**	19](#_Toc220160263)

[**Fig. S3.**	20](#_Toc220160264)

**Section S1: Methods**

**S1.1 Unit normalization**

We use the equation below to normalize the different units in our dataset:

$${\%RPA}_{k}= \frac{\left( \frac{PEU}{MW} \right)_{k}}{\sum_{i=1}^{n} \left( \frac{PEU}{MW} \right)_{i}}\times100$$

Where ${\%RPA}_{k}$ is %-Relative Protein Abundance for protein *k*, PEU is Protein Expression Unit (i.e., the unit reported by the original article for protein expression levels, such as spectral count, iBAQ), and MW is the Molecular Weight of protein *k*. In this equation, the molecular weight of the protein is normalized by the reported abundance and expressed as a percentage of the total protein corona reported.

**S1.2 Performance metrics for adsorption classification model**

The adsorption classification model was evaluated using six standard performance metrics: accuracy, precision, recall (sensitivity), F1 score, area under the receiver operating characteristic curve (AUC), and Matthews correlation coefficient (MCC). For each protein-specific classifier, predictions were compared against binary ground-truth labels indicating adsorption (1) or non-adsorption (0), and the following metrics were computed:

(a) **Accuracy**

$$Accuracy= \frac{\mathrm{TP}+TN}{TP+TN+FP+FN}$$

(b) **Precision**

$$Precision= \frac{\mathrm{TP}}{TP+FP}$$

(c) **Recall (Sensitivity)**

$$Recall= \frac{\mathrm{TP}}{TP+FN}$$

(d) **F1 Score**

$$F1= 2 \times\frac{Precision \times Recall}{Precision+Recall}$$

(e) **AUC (Area Under ROC curve)**

$$AUC= \int TPR d(FPR)$$

(f) **MCC (Matthew’s Correlation Coefficient)**

$$MCC= \frac{TP \times TN-FP\times FN}{\sqrt{(TP+FP)(TP+FN)(TN+FP)(TN+FN)}}$$

Where TP is True Positive, TN is True Negative, FP is False Positive, FN is False Negative, TPR is the True Positive prediction Rate, and FPR is the False Positive prediction Rate. All classification metrics were first computed independently for each protein using its corresponding test samples. Reported per-protein and category-level values represent the mean ± standard deviation across proteins, thereby weighing each protein equally regardless of sample size. In addition, to visualize global classification behavior, predicted probabilities and labels were concatenated across all proteins to generate pooled ROC curves and confusion matrices, which reflect sample-weighted overall model discrimination.

**S1.3 Performance metrics for abundance regression Model**

Protein abundance regression performance was evaluated using the coefficient of determination ($R^{2}$), mean squared error (MSE), root mean squared error (RMSE), and mean absolute error (MAE). All metrics were computed on the log10-transformed abundance scale to ensure stable evaluation across multiple orders of magnitude. Only samples with positive observed and predicted abundance values were included in the analysis.

**S1.3.1 Per-protein regression metrics**

For each protein $k$ with $n_{k}$valid samples, regression performance was evaluated independently. The coefficient of determination was calculated as:

$$R_{k}^{2}= 1-\frac{\sum_{j = 1}^{n_{k}} {(z_{kj}- \hat{z}_{kj})}^{2}}{\sum_{j = 1}^{n_{k}} {(z_{kj}- \bar{z}_{k})}^{2}}$$

where $z_{kj}=\log_{10}(y_{kj})$is the observed abundance, $\hat{z}_{kj}=\log_{10}(\hat{y}_{kj})$is the predicted abundance, and $\bar{z}_{k}$is the mean of the observed log-abundances for protein $k$.

The corresponding error metrics were computed as:

$\mathrm{MSE}_{k}= \frac{1}{n_{k}} \sum_{j=1}^{n_{k}} {(z_{kj}- \hat{z}_{kj})}^{2}$, $\mathrm{RMSE}_{k}=\sqrt{\mathrm{MSE}_{k}}$, $\mathrm{MAE}_{k}= \frac{1}{n_{k}} \sum_{j=1}^{n_{k}} |z_{kj}- \hat{z}_{kj}|$

Per-protein regression metrics were subsequently summarized as mean ± standard deviation across proteins, both overall and within functional protein categories.

**S1.3.2 Pooled regression performance**

To quantify overall model accuracy across the entire protein set, **pooled regression metrics** were additionally computed. For pooled evaluation, predicted and observed abundance values from all proteins were concatenated into a single combined dataset, yielding a total of $N_{\text{tot}}$protein–sample pairs. The pooled coefficient of determination was calculated as:

$$R_{pooled}^{2}=1-\frac{\sum_{k,j} {{(z}_{kj}-\hat{z}_{kj})}^{2}}{\sum_{k,j} {{(z}_{kj}-\bar{z})}^{2}},$$

where $\bar{z}$ denotes the global mean of all observed log-abundances. Pooled RMSE, MAE, and MSE were computed analogously over the combined dataset. Pooled metrics provide a sample-weighted global measure of regression performance across all protein-NP pairs and are reported alongside per-protein and category-level metrics to highlight differences between overall accuracy and protein-specific heterogeneity.

Together, pooled, per-protein, and category-level evaluations provide complementary perspectives on model performance: pooled metrics reflect overall predictive accuracy across all samples, while per-protein and category-level metrics characterize variability and functional specificity in protein corona adsorption and abundance prediction. Performance summaries for both classification and regression models are reported in Tables 1-2.

**S1.4 Standardized Global Residuals for Error Analysis**

To support applicability domain (AD) diagnostics, standardized global residuals were computed for each prediction:

$$r_{t}=\frac{y-\hat{y}}{{SD}_{train residuals}}$$

Residuals with $\mid r_{t}\mid>3$were considered extreme outliers. These values were used in combination with leverage values (for regression) and *k*-nearest neighbor distance (for classification) to identify predictions outside the model's applicability domain.

**S1.5 Feature Importance Sanity Check**

To assess whether SHAP-derived feature importance reflects observable data patterns rather than model artifacts, we performed a stratified sanity-check analysis on representative top features (np_subtype, mod_type, and iso_time_min). For each feature, the test set was partitioned based on feature type: categorical levels were used for one-hot encoded variables (e.g., silica, metal, lipid for np_subtype), while continuous variables (e.g., iso_time_min) were divided into tertiles (low, medium, high). For each protein, feature-associated separation was quantified by computing the difference between the maximum and minimum group-level outcome values. For the classification model, the outcome was defined as the adsorption rate (proportion of samples with adsorption = 1), whereas for the regression model, it was defined as the mean test-set abundance. These differences were then summarized across proteins using both mean and median statistics.

The classification results (Table S8) show that np_subtype produces the largest separation in adsorption rate (mean Δ = 0.76, median = 0.75), followed by mod_type (mean = 0.64, median = 0.60) and iso_time_min (mean = 0.25, median = 0.26). The regression results (Table S9) show the same ordering of feature importance. This consistent ranking across both tasks aligns with the SHAP-derived feature importance reported in the main analysis, supporting that model-derived feature contributions reflect underlying data patterns. Notably, the regression distributions are right-skewed (e.g., for np_subtype: mean = 0.98 vs. median = 0.25), indicating that while most proteins exhibit moderate abundance differences across nanoparticle subtypes, a subset of proteins shows substantially higher sensitivity, consistent with the heterogeneous feature contributions observed in the SHAP heatmap (Fig. 6A).

**Section S2: Supplementary Tables**

**Table S1.**

Nanoparticle Physicochemical Properties and Experimental Conditions Used for Modeling

| **Abbreviation** | **Full Name** | **Value/Description** |
| --- | --- | --- |
| mod_type | Surface Modification Type | No modification; Polymer; Alkylthiol; Carboxylic acid; Amine-based; Amino acid; Lipid; Surfactant; Other |
| mod_charge | Surface Modification Charge | Positive; Neutral; Negative; Unmodified |
| np_type | Nanoparticle Material Type | Organic; Inorganic |
| np_subtype | Nanoparticle Material Subtype | Metal; Metal oxide; Silica; Polystyrene; Lipid; Niosome; DNA/Latex; Other |
| size_nm | Particle Size (nm) | Numerical variable: binned categories include: <50 nm; 50-100 nm; 100-150 nm; >150 nm |
| zeta_mv | Zeta Potential (mV) | Continuous numeric values (e.g., -80 to +40 mV) |
| zeta_charge | Zeta Potential Charge Category | Positive; Neutral (-10 to +10 mV); Negative |
| incub_time_min | Incubation Time (min) | <30 min; 30-60 min; >60 min |
| agitation | Agitation During Incubation | Yes; No |
| iso_time_min | Isolation Time (min) | <30 min; 30-60 min; >60 min |
| wash_steps | Number of Wash Steps | 0; 1; 2; 3; ≥4 (majority between 1-3) |

**Table S2.**

Functional Categorization of the 285 Proteins Included in the Adsorption Classification Model

| **Category** | **N** | **Protein** |
| --- | --- | --- |
| Apolipoproteins | 15 | Apolipoprotein A-I; Apolipoprotein A-II; Apolipoprotein A-IV; Apolipoprotein A-V; Apolipoprotein B-100; Apolipoprotein C-I; Apolipoprotein C-II; Apolipoprotein C-III; Apolipoprotein C-IV; Apolipoprotein D; Apolipoprotein E; Apolipoprotein F; Apolipoprotein L1; Apolipoprotein M; Apolipoprotein(a) |
| Coagulation/Fibrinogen | 10 | Coagulation factor IX; Coagulation factor V; Coagulation factor VII; Coagulation factor X; Coagulation factor XI; Coagulation factor XII; Coagulation factor XIII A chain; Fibrinogen alpha chain; Fibrinogen beta chain; Fibrinogen gamma chain |
| Complement System | 27 | C4b-binding protein alpha chain; C4b-binding protein beta chain; Complement C1q subcomponent subunit A; Complement C1q subcomponent subunit B; Complement C1q subcomponent subunit C; Complement C1r subcomponent; Complement C1r subcomponent-like protein; Complement C1s subcomponent; Complement C2; Complement C3; Complement C4-A; Complement C4-B; Complement C5; Complement component C6; Complement component C7; Complement component C8 alpha chain; Complement component C8 beta chain; Complement component C8 gamma chain; Complement component C9; Complement factor B; Complement factor D; Complement factor H; Complement factor H-related protein 1; Complement factor H-related protein 2; Complement factor H-related protein 3; Complement factor H-related protein 5; Complement factor I |
| Cytoskeletal | 40 | Actin cytoplasmic 1; Actin cytoplasmic 2; Actin gamma-enteric smooth muscle; Alpha-actinin-1; Alpha-actinin-4; Beta actin like protein 2; Filamin-A; Keratin, type I cuticular Ha1; Keratin, type I cuticular Ha7; Keratin, type I cytoskeletal 10; Keratin, type I cytoskeletal 14; Keratin, type I cytoskeletal 15; Keratin, type I cytoskeletal 16; Keratin, type I cytoskeletal 17; Keratin, type I cytoskeletal 18; Keratin, type I cytoskeletal 19; Keratin, type I cytoskeletal 20; Keratin, type I cytoskeletal 24; Keratin, type I cytoskeletal 26; Keratin, type I cytoskeletal 28; Keratin, type I cytoskeletal 9; Keratin, type II cytoskeletal 1; Keratin, type II cytoskeletal 1b; Keratin, type II cytoskeletal 2 epidermal; Keratin, type II cytoskeletal 2 oral; Keratin, type II cytoskeletal 4; Keratin, type II cytoskeletal 5; Keratin, type II cytoskeletal 6A; Keratin, type II cytoskeletal 6B; Keratin, type II cytoskeletal 7; Keratin, type II cytoskeletal 74; Keratin, type II cytoskeletal 79; Myosin-10; Myosin-11; Myosin-9; Programmed cell death 6-interacting protein; Prolactin inducible protein; Tropomyosin alpha-4 chain; Tubulin alpha-1C chain; Tubulin alpha-4A chain |
| Immunoglobulins | 41 | Immunoglobulin J chain; Immunoglobulin heavy constant alpha 1; Immunoglobulin heavy constant alpha 2; Immunoglobulin heavy constant delta; Immunoglobulin heavy constant gamma 1; Immunoglobulin heavy constant gamma 2; Immunoglobulin heavy constant gamma 3; Immunoglobulin heavy constant gamma 4; Immunoglobulin heavy constant mu; Immunoglobulin heavy variable 1-3; Immunoglobulin heavy variable 1-46; Immunoglobulin heavy variable 3-13; Immunoglobulin heavy variable 3-23 ; Immunoglobulin heavy variable 3-48 ; Immunoglobulin heavy variable 3-53 ; Immunoglobulin heavy variable 3-7; Immunoglobulin heavy variable 4-34; Immunoglobulin heavy variable 4-39; Immunoglobulin heavy variable 4-59; Immunoglobulin kappa constant; Immunoglobulin kappa light chain; Immunoglobulin kappa variable 1-13; Immunoglobulin kappa variable 1-16; Immunoglobulin kappa variable 1-17; Immunoglobulin kappa variable 1-5; Immunoglobulin kappa variable 1D-12; Immunoglobulin kappa variable 1D-33; Immunoglobulin kappa variable 2D-28; Immunoglobulin kappa variable 3-11; Immunoglobulin kappa variable 3-15; Immunoglobulin kappa variable 3-20; Immunoglobulin kappa variable 4-1; Immunoglobulin lambda constant 1; Immunoglobulin lambda constant 2; Immunoglobulin lambda constant 3; Immunoglobulin lambda constant 7; Immunoglobulin lambda variable 1-47; Immunoglobulin lambda variable 1-51; Immunoglobulin lambda variable 3-19; Immunoglobulin lambda variable 3-21; Immunoglobulin lambda-like polypeptide 5; |
| Metabolic Enzymes | 8 | Glutathione peroxidase 3; Glyceraldehyde-3-phosphate dehydrogenase; Peptidyl-prolyl cis-trans isomerase A; Phosphatidylcholine-sterol acyltransferase; Phosphoglycerate kinase 1; Prenylcysteine oxidase 1; Pyruvate kinase PKM; Sulfhydryl oxidase 1 |
| Protease/Inhibitors | 5 | Mannan-binding lectin serine protease 1; Mannan-binding lectin serine protease 2; Plasma protease C1 inhibitor; Plasma serine protease inhibitor; Protein Z-dependent protease inhibitor |
| Transport/Binding | 15 | Galectin-3-binding protein; Haptoglobin; Haptoglobin-related protein; Hyaluronan-binding protein 2; Insulin-like growth factor-binding protein 2; Insulin-like growth factor-binding protein 3; Insulin-like growth factor-binding protein complex acid labile chain; Lactotransferrin; Latent-transforming growth factor beta-binding protein 1; Lipopolysaccharide-binding protein; Mannose-binding protein C; Retinol-binding protein 4; Serotransferrin; Serum albumin; Vitamin D-binding protein |
| Other/Mixed | 124 | 14-3-3 protein epsilon; 14-3-3 protein sigma; 14-3-3 protein zeta/delta; 78kDa glucose-regulated protein; Adenylyl cyclase-associated protein 1; Afamin; Alpha-1-acid glycoprotein 1; Alpha-1-acid glycoprotein 2; Alpha-1-antichymotrypsin; Alpha-1-antitrypsin; Alpha-1B-glycoprotein; Alpha-2-HS-glycoprotein; Alpha-2-antiplasmin; Alpha-2-macroglobulin; Alpha-enolase; Amyloid beta A4 protein; Angiogenin; Angiopoietin related protein 3; Angiotensinogen; Anthrax toxin receptor 2; Antithrombin-III; Band 3 anion transport protein; Beta enolase; Beta-2-glycoprotein 1; C-reactive protein; CD5 antigen-like; Calsyntenin 1; Carboxypeptidase B2; Carboxypeptidase N catalytic chain; Carboxypeptidase N subunit 2; Cartilage acidic protein 1; Cartilage oligomeric matrix protein; Cathelicidin antimicrobial peptide; Centrosomal protein of 162 kDa; Ceruloplasmin; Cholesteryl ester transfer protein; Clusterin; Cofilin-1; Coiled coil domain containing protein 144B; Cystatin-C; Dermcidin; Extracellular matrix protein 1; Fermitin family homolog 3; Fibronectin; Fibulin-1; Ficolin-2; Ficolin-3; Flavin reductase (NADPH); Fructose-bisphosphate aldolase A; Gelsolin; Heat shock cognate 71 kDa protein; Heat shock related 70 kDa protein 2; Hemoglobin subunit alpha; Hemoglobin subunit beta; Hemoglobin subunit delta; Hemopexin; Heparin cofactor 2; Hepatocyte growth factor activator; Hepatocyte growth factor-like protein; Histidine-rich glycoprotein; Histone H4; Inactive cell surface hyaluronidase CEMIP2; Integrin alpha-IIb; Integrin beta-3; Inter-alpha-trypsin inhibitor heavy chain H1; Inter-alpha-trypsin inhibitor heavy chain H2; Inter-alpha-trypsin inhibitor heavy chain H4; Inter-alpha-trypsin inhibitor, heavy chain H3; Kallistatin; Kininogen-1; Lumican; Lysozyme C; Moesin; Multimerin-1; N-acetylmuramoyl-L-alanine amidase; Nebulin related anchoring protein; Neurofilament heavy polypeptide; POTE ankyrin domain family member F; POTE ankyrin domain family member J; Phosphatidylinositol-glycan specific phospholipase D; Phospholipid transfer protein; Pigment epithelium-derived factor; Plasma kallikrein; Plasminogen; Plasminogen activator inhibitor 1; Platelet basic protein; Platelet factor 4; Platelet factor 4 variant; Pregnancy zone protein; Procollagen C-endopeptidase enhancer 1; Profilin-1; Properdin; Protein AMBP; Protein S100-A7; Protein S100-A9; Proteoglycan 4; Prothrombin; Ras-related protein Rap-1b; Secreted phosphoprotein 24; Selenoprotein P; Serum amyloid A-1 protein; Serum amyloid A-4 protein; Serum amyloid P-component; Serum paraoxonase lactonase 3; Serum paraoxonase/arylesterase 1; Soluble scavenger receptor cysteine-rich domain-containing protein SSC5D; Stomatin; TBC1 domain family member 2A; Talin-1; Tetranectin; Thrombospondin-1; Thrombospondin-2; Thrombospondin-4; Transaldolase; Transthyretin; Ubiquitin-ribosomal protein eS31 fusion protein; Vinculin; Vitamin K-dependent protein C; Vitamin K-dependent protein S; Vitamin K-dependent protein Z; Vitronectin; Zinc-alpha-2-glycoprotein; von Willebrand factor; Transforming growth factor-beta-induced protein ig-h3; Transforming growth factor-beta-induced protein ig-h3 |

**Table S3.**

Functional Categorization of the 220 Proteins Included in the Abundance Regression Model

| **Category** | **N** | **Protein** |
| --- | --- | --- |
| Apolipoproteins | 15 | Apolipoprotein A-I; Apolipoprotein A-II; Apolipoprotein A-IV; Apolipoprotein A-V; Apolipoprotein B-100; Apolipoprotein C-I; Apolipoprotein C-II; Apolipoprotein C-III; Apolipoprotein C-IV; Apolipoprotein D; Apolipoprotein E; Apolipoprotein F; Apolipoprotein L1; Apolipoprotein M; Apolipoprotein(a) |
| Coagulation/Fibrinogen | 11 | Coagulation factor IX; Coagulation factor V; Coagulation factor VII; Coagulation factor X; Coagulation factor XI; Coagulation factor XII; Coagulation factor XIII A chain; Fibrinogen alpha chain; Fibrinogen beta chain; Fibrinogen gamma chain; Tissue Factor |
| Complement System | 23 | C4b-binding protein alpha chain; C4b-binding protein beta chain; Complement C1q subcomponent subunit A; Complement C1q subcomponent subunit B; Complement C1q subcomponent subunit C; Complement C1r subcomponent; Complement C1s subcomponent; Complement C3; Complement C4-A; Complement C4-B; Complement C5; Complement component C6; Complement component C7; Complement component C8 alpha chain; Complement component C8 beta chain; Complement component C8 gamma chain; Complement component C9; Complement factor B; Complement factor D; Complement factor H; Complement factor H-related protein 1; Complement factor H-related protein 5; Complement factor I |
| Cytoskeletal | 20 | Actin alpha cardiac muscle 1; Actin aortic smooth muscle; Actin cytoplasmic 1; Actin cytoplasmic 2; Alpha-actinin-1; Filamin-A; Keratin, type I cytoskeletal 10; Keratin, type I cytoskeletal 14; Keratin, type I cytoskeletal 16; Keratin, type I cytoskeletal 19; Keratin, type I cytoskeletal 9; Keratin, type II cytoskeletal 1; Keratin, type II cytoskeletal 1b; Keratin, type II cytoskeletal 2 epidermal; Keratin, type II cytoskeletal 5; Keratin, type II cytoskeletal 6A; Keratin, type II cytoskeletal 6C; Myosin-9; Tropomyosin alpha-4 chain; Tubulin alpha-4A chain |
| Immunoglobulins | 26 | Immunoglobulin J chain; Immunoglobulin heavy constant alpha 1; Immunoglobulin heavy constant alpha 2; Immunoglobulin heavy constant gamma 1; Immunoglobulin heavy constant gamma 2; Immunoglobulin heavy constant gamma 3; Immunoglobulin heavy constant gamma 4; Immunoglobulin heavy constant mu; Immunoglobulin heavy variable 3-13; Immunoglobulin heavy variable 3-23 ; Immunoglobulin heavy variable 3-7; Immunoglobulin heavy variable 5-51; Immunoglobulin kappa constant; Immunoglobulin kappa light chain; Immunoglobulin kappa variable 1-17; Immunoglobulin kappa variable 1D-33; Immunoglobulin kappa variable 2D-28; Immunoglobulin kappa variable 3-11; Immunoglobulin kappa variable 3-15; Immunoglobulin kappa variable 3-20; Immunoglobulin kappa variable 4-1; Immunoglobulin lambda constant 2; Immunoglobulin lambda constant 3; Immunoglobulin lambda variable 1-47; Immunoglobulin lambda variable 3-21; Immunoglobulin lambda-like polypeptide 5 |
| Metabolic Enzymes | 7 | Glutathione S-transferase P; Glutathione peroxidase 3; Glyceraldehyde-3-phosphate dehydrogenase; Peptidyl-prolyl cis-trans isomerase A; Peptidyl-prolyl cis-trans isomerase B; Pyruvate kinase PKM; Sulfhydryl oxidase 1 |
| Protease/Inhibitors | 6 | Cathepsin D; Mannan-binding lectin serine protease 1; Plasma protease C1 inhibitor; Plasma serine protease inhibitor; Protein Z-dependent protease inhibitor; Serpin B3 |
| Transport/Binding | 14 | Galectin-3-binding protein; Haptoglobin; Haptoglobin-related protein; Hyaluronan-binding protein 2; Insulin-like growth factor-binding protein 2; Insulin-like growth factor-binding protein 3; Insulin-like growth factor-binding protein 5; Insulin-like growth factor-binding protein complex acid labile chain; Lactotransferrin; Lipopolysaccharide-binding protein; Retinol-binding protein 4; Serotransferrin; Serum albumin; Vitamin D-binding protein |
| Other/Mixed | 98 | 14-3-3 protein zeta/delta; Afamin; Alpha-1-acid glycoprotein 1; Alpha-1-acid glycoprotein 2; Alpha-1-antichymotrypsin; Alpha-1-antitrypsin; Alpha-1B-glycoprotein; Alpha-2-HS-glycoprotein; Alpha-2-antiplasmin; Alpha-2-macroglobulin; Alpha-enolase; Angiogenin; Angiotensinogen; Annexin A2; Antithrombin-III; Band 3 anion transport protein; Beta-2-glycoprotein 1; C-reactive protein; CD5 antigen-like; Carboxypeptidase B2; Carboxypeptidase N catalytic chain; Cartilage acidic protein 1; Cartilage oligomeric matrix protein; Centrosomal protein of 162 kDa; Ceruloplasmin; Cholesteryl ester transfer protein; Clusterin; Cofilin-1; Dermcidin; Desmoplakin; Elongation factor 1-alpha 1; Elongation factor 2; Fermitin family homolog 3; Fibronectin; Fibulin-1; Ficolin-3; Fructose-bisphosphate aldolase A; Gelsolin; Heat shock cognate 71 kDa protein; Heat shock protein beta-1; Hemoglobin subunit alpha; Hemoglobin subunit beta; Hemopexin; Heparin cofactor 2; Hepatocyte growth factor activator; Histidine-rich glycoprotein; Histone H4; Hornerin; Integrin alpha-IIb; Integrin beta-3; Inter-alpha-trypsin inhibitor heavy chain H1; Inter-alpha-trypsin inhibitor heavy chain H2; Inter-alpha-trypsin inhibitor heavy chain H4; Inter-alpha-trypsin inhibitor, heavy chain H3; Kininogen-1; Lumican; Lysozyme C; Moesin; Multimerin-1; N-acetylmuramoyl-L-alanine amidase; Phospholipid transfer protein; Pigment epithelium-derived factor; Plasma kallikrein; Plasminogen; Platelet basic protein; Platelet factor 4; Platelet factor 4 variant; Pleckstrin; Pregnancy zone protein; Profilin-1; Properdin; Protein AMBP; Protein S100-A8; Protein S100-A9; Proteoglycan 4; Prothrombin; Ras-related protein Rap-1b; Secreted phosphoprotein 24; Selenoprotein P; Serum amyloid A-1 protein; Serum amyloid A-4 protein; Serum amyloid P-component; Serum paraoxonase/arylesterase 1; Stomatin; Talin-1; Tetranectin; Thrombospondin-1; Thrombospondin-2; Thrombospondin-4; Transthyretin; Ubiquitin-ribosomal protein eS31 fusion protein; Vinculin; Vitamin K-dependent protein C; Vitamin K-dependent protein S; Vitamin K-dependent protein Z; Vitronectin; Zinc-alpha-2-glycoprotein; von Willebrand factor |

**Table S4.**

Adsorption Classification Model Feature Importance Sanity Check

| **Top 3 Features** | **Mean Δrate** | **Median Δrate** |
| --- | --- | --- |
| np_subtype | 0.76 | 0.75 |
| mod_type | 0.64 | 0.60 |
| iso_time_min | 0.25 | 0.26 |

**Table S5.**

Abundance Regression Model Feature Importance Sanity Check

|  | **Mean Δabundance** | **Median Δabundance** |
| --- | --- | --- |
| **np_subtype** | 0.98 | 0.25 |
| **mod_type** | 0.83 | 0.26 |
| **iso_time_min** | 0.32 | 0.09 |

**Table S6.**

Comparison of DNN, Random Forest (RF), XGBoost, Logistic Regression (LR) Adsorption Classification Model Performance

|  | Accuracy | F1 Score | Precision | Recall | AUC | MCC |
| --- | --- | --- | --- | --- | --- | --- |
| *DNN* |  |  |  |  |  |  |
| Training | 0.93 ± 0.03 | 0.91 ± 0.04 | 0.87 ± 0.08 | 0.95 ± 0.04 | 0.98 ± 0.01 | 0.84 ± 0.06 |
| Test | 0.90 ± 0.04 | 0.86 ± 0.06 | 0.82 ± 0.10 | 0.91 ± 0.06 | 0.96 ± 0.03 | 0.76 ± 0.09 |
| *RF* |  |  |  |  |  |  |
| Training | 0.92 ± 0.04 | 0.89 ± 0.08 | 0.90 ± 0.07 | 0.89 ± 0.10 | 0.98 ± 0.02 | 0.81 ± 0.09 |
| Test | 0.89 ± 0.05 | 0.85 ± 0.10 | 0.84 ± 0.10 | 0.87 ± 0.14 | 0.96 ± 0.03 | 0.74 ± 0.12 |
| *XGBoost* |  |  |  |  |  |  |
| Training | 0.85 ± 0.11 | 0.81 ± 0.11 | 0.80 ± 0.14 | 0.86 ± 0.15 | 0.93 ± 0.08 | 0.70 ± 0.17 |
| Test | 0.82 ± 0.11 | 0.77 ± 0.11 | 0.74 ± 0.16 | 0.83 ± 0.16 | 0.91 ± 0.09 | 0.62 ± 0.17 |
| *LR* |  |  |  |  |  |  |
| Training | 0.90 ± 0.05 | 0.86 ± 0.09 | 0.83 ± 0.12 | 0.92 ± 0.06 | 0.96 ± 0.03 | 0.77 ± 0.11 |
| Test | 0.87 ± 0.05 | 0.83 ± 0.09 | 0.80 ± 0.14 | 0.90 ± 0.08 | 0.95 ± 0.04 | 0.71 ± 0.11 |

**Table S7.**

Comparison of DNN, Random Forest (RF), XGBoost, Ridge Regression (RR) Abundance Regression Model Performance

|  | R^2^ | RMSE | MAE | MSE |
| --- | --- | --- | --- | --- |
| *DNN* |  |  |  |  |
| Training | 0.63 ± 0.16 | 0.47 ± 0.25 | 0.33 ± 0.19 | 0.28 ± 0.26 |
| Test | 0.40 ± 0.33 | 0.61 ± 0.27 | 0.43 ± 0.22 | 0.45 ± 0.35 |
| *RF* |  |  |  |  |
| Training | 0.44 ± 0.28 | 0.62 ± 1.02 | 0.21 ± 0.39 | 1.43 ± 4.98 |
| Test | 0.20 ± 0.42 | 0.69 ± 1.19 | 0.27 ± 0.46 | 1.89 ± 8.06 |
| *XGBoost* |  |  |  |  |
| Training | 0.16 ± 0.17 | 0.80 ± 1.33 | 0.29 ± 0.53 | 2.39 ± 8.61 |
| Test | 0.09 ± 0.20 | 0.78 ± 1.30 | 0.30 ± 0.53 | 2.27 ± 8.50 |
| *LR* |  |  |  |  |
| Training | 0.18 ± 0.20 | 0.76 ± 1.25 | 0.29 ± 0.51 | 2.15 ± 7.38 |
| Test | -0.47 ± 5.99 | 0.84 ± 1.48 | 0.32 ± 0.56 | 2.88 ± 11.49 |

**Table S8.**

Adsorption Classification Model Performance for Sensitivity Analysis

|  | **Protein Retained** | **Accuracy** | **F1 Score** | **Precision** | **Recall** | **AUC** | **MCC** |
| --- | --- | --- | --- | --- | --- | --- | --- |
| **1%** | 636 | 0.94 ± 0.05 | 0.76 ± 0.20 | 0.75 ± 0.23 | 0.84 ± 0.23 | 0.93 ± 0.13 | 0.73 ± 0.18 |
| **5%** | 285 | 0.90 ± 0.05 | 0.86 ± 0.07 | 0.82 ± 0.11 | 0.91 ± 0.07 | 0.96 ± 0.03 | 0.76 ± 0.09 |
| **10%** | 276 | 0.89 ± 0.05 | 0.86 ± 0.07 | 0.83 ± 0.11 | 0.91 ± 0.07 | 0.96 ± 0.03 | 0.76 ± 0.10 |

**Table S9.**

Abundance Regression Model Performance for Sensitivity Analysis

|  | **Protein Retained** | **R^2^** | **RMSE** | **MAE** | **MSE** |
| --- | --- | --- | --- | --- | --- |
| **1%** | 727 | -1.01 ± 7.84 | 0.26 ± 0.28 | 0.18 ± 0.21 | 0.15 ± 0.28 |
| **5%** | 220 | 0.40 ± 0.33 | 0.61 ± 0.27 | 0.43 ± 0.22 | 0.45 ± 0.35 |
| **10%** | 173 | 0.41 ± 0.22 | 0.70 ± 0.22 | 0.50 ± 0.19 | 0.53 ± 0.34 |

**Section S3: Supplementary Figures**

**Fig. S1.**

Workflow for protein corona database curation and model development

**Fig. S2.**

Precision–recall (PR) curves for the adsorption classification model under class imbalance.

PR curves are shown for all test samples (Grey), in-applicability-domain (in-AD, blue), and out-of-applicability-domain (out-of-AD, red) subsets. Shaded regions represent bootstrap-derived confidence intervals. The model achieves high average precision (AP = 0.945 overall), with improved performance for in-AD samples (AP = 0.964) compared to out-of-AD samples (AP = 0.844). The dashed horizontal line indicates the baseline corresponding to the positive class prevalence.

**Fig. S3.**

Distribution of per-protein test R^2^ values for abundance regression models.

Histogram showing the distribution of R^2^ values across all protein-specific regression models. The dashed vertical line indicates R^2^ = 0. In a total of 220 proteins, 147 proteins (Blue) achieved positive predictive performance (R^2^ ≥ 0), while 73 proteins (Red) exhibited negative R^2^ values, reflecting variability in model performance across proteins.

**Fig. S4.**

Learning curve analysis of abundance regression model performance.

Mean R^2^ across proteins is plotted as a function of training set size (20–100%), for both training (blue) and test (orange) sets. Shaded regions represent 95% confidence intervals.

**Fig. S5.**

Signed SHAP heatmap for the adsorption classification model. The y-axis lists the top 30 proteins, and the x-axis lists the 11 input features ranked by overall mean |SHAP| value. Each cell represents the mean signed SHAP value of a given feature for a given protein, computed by averaging signed SHAP values across all test-set samples of that protein's model. Red indicates a positive contribution (increasing the predicted probability of adsorption), blue indicates a negative contribution (decreasing the predicted probability), and white indicates near-zero or balanced bidirectional contributions.

**Fig. S6.**

Sub-feature SHAP analysis for one-hot-encoded categorical variables in the classification model. Each panel decomposes a parent categorical variable into its constituent dummy variables and displays both the mean |SHAP| value (top x-axis, horizontal bars) and the SHAP value distribution across samples (bottom x-axis, scatter points). Numerical labels on the bars denote the mean |SHAP| value and the percentage contribution of each dummy variable to its parent categorical variable. Scatter points are colored by feature value (red = 1, blue = 0), where a value of 1 indicates that the sample belongs to that specific category. (A) np_subtype, decomposed into eight NP subtypes. (B) mod_type, decomposed into thirteen surface modification categories. (C) np_type, decomposed into Inorganic and Organic. (D) mod_charge, decomposed into No Modification, Positive, Neutral, and Negative. (E) zeta_charge, decomposed into Neutral, Positive, and Negative. (F) agitation, decomposed into N (no agitation) and Y (with agitation). Green shading indicates NP physicochemical properties; purple shading indicates experimental conditions.

**Fig. S7.**

Signed SHAP heatmap for the abundance regression model. The y-axis lists the top 30 proteins, and the x-axis lists the 11 input features ranked by overall mean |SHAP| value. Each cell represents the mean signed SHAP value of a given feature for a given protein, computed by averaging signed SHAP values across all test-set samples of that protein's model. Red indicates a positive contribution (increasing the predicted probability of adsorption), blue indicates a negative contribution (decreasing the predicted probability), and white indicates near-zero or balanced bidirectional contributions.

**Fig. S8.**

Sub-feature SHAP analysis for one-hot-encoded categorical variables in the regression model. Each panel decomposes a parent categorical variable into its constituent dummy variables and displays both the mean |SHAP| value (top x-axis, horizontal bars) and the SHAP value distribution across samples (bottom x-axis, scatter points). Numerical labels on the bars denote the mean |SHAP| value and the percentage contribution of each dummy variable to its parent categorical variable. Scatter points are colored by feature value (red = 1, blue = 0), where a value of 1 indicates that the sample belongs to that specific category. (A) np_subtype, decomposed into eight NP subtypes. (B) mod_type, decomposed into thirteen surface modification categories. (C) np_type, decomposed into Inorganic and Organic. (D) mod_charge, decomposed into No Modification, Positive, Neutral, and Negative. (E) zeta_charge, decomposed into Neutral, Positive, and Negative. (F) agitation, decomposed into N (no agitation) and Y (with agitation). Green shading indicates NP physicochemical properties; purple shading indicates experimental conditions.

**Fig. S9.**

Combined feature importance and SHAP scatter plot for all features (with dose information) in adsorption classification models.

**Fig. S10.**

Combined feature importance and SHAP scatter plot for all features (with dose information) in abundance regression models.
